# Codon optimization and improved delivery/immunization regimen enhance the immune response against wild-type and drug-resistant HIV-1 reverse transcriptase, preserving its Th2-polarity

**DOI:** 10.1038/s41598-018-26281-z

**Published:** 2018-05-24

**Authors:** A. A. Latanova, S. Petkov, A. Kilpelainen, J. Jansons, O. E. Latyshev, Y. V. Kuzmenko, J. Hinkula, M. A. Abakumov, V. T. Valuev-Elliston, M. Gomelsky, V. L. Karpov, F. Chiodi, B. Wahren, D. Y. Logunov, E. S. Starodubova, M. G. Isaguliants

**Affiliations:** 1 Engelhardt Institute of Molecular Biology, Russian Academy of Sciences, Moscow, Russia; 2 Department of Microbiology, Tumor and Cell Biology, Karolinska Institute, Stockholm, Sweden; 3 Gamaleja Research Center of Epidemiology and Microbiology, Moscow, Russia; 4 Chumakov Federal Scientific Center for Research and Development of Immune-and- Biological Products of the Russian Academy of Sciences, Moscow, Russia; 5 Riga Stradins University, Riga, Latvia; 6 Linköping University, Linköping, Sweden; 7 Research and Education Center for Medical Nanobiotechnology, Pirogov Russian National Research Medical University, Ministry of Health of the Russian Federation, Moscow, Russia; 8 National University of Science and Technology (MISIS), Moscow, Russia; 9 Department of Molecular Biology, University of Wyoming, Laramie, WY 82071 USA

## Abstract

DNA vaccines require a considerable enhancement of immunogenicity. Here, we optimized a prototype DNA vaccine against drug-resistant HIV-1 based on a weak Th2-immunogen, HIV-1 reverse transcriptase (RT). We designed expression-optimized genes encoding inactivated wild-type and drug-resistant RTs (RT-DNAs) and introduced them into mice by intradermal injections followed by electroporation. RT-DNAs were administered as single or double primes with or without cyclic-di-GMP, or as a prime followed by boost with RT-DNA mixed with a luciferase-encoding plasmid (“surrogate challenge”). Repeated primes improved cellular responses and broadened epitope specificity. Addition of cyclic-di-GMP induced a transient increase in IFN-γ production. The strongest anti-RT immune response was achieved in a prime-boost protocol with electroporation by short 100V pulses done using penetrating electrodes. The RT-specific response, dominated by CD4+ T-cells, targeted epitopes at aa 199–220 and aa 528–543. Drug-resistance mutations disrupted the epitope at aa 205–220, while the CTL epitope at aa 202–210 was not affected. Overall, multiparametric optimization of RT strengthened its Th2- performance. A rapid loss of RT/luciferase-expressing cells in the surrogate challenge experiment revealed a lytic potential of anti-RT response. Such lytic CD4+ response would be beneficial for an HIV vaccine due to its comparative insensitivity to immune escape.

## Introduction

HIV evolution with acquisition of new mutations leads to the continuous emergence of the (multi)drug-resistant HIV strains necessitating development of new anti-retroviral drugs. It has been proposed that anti-viral immune response can induce a selection pressure on the virus, shape viral strains circulating in different groups of the population, and control viral load in a minority of HIV-infected individuals (elite controllers)^1–5^. An anti-viral immune response against mutations that confer drug resistance might thus limit viral evolution towards resistant phenotypes leading to a more effective antiretroviral therapy (ART)^6–8^.

However, in most cases of successful ART there is no antigen stimulation, and this leads to a gradual loss of the anti-HIV immune response and limits the possibilities of immune control. The idea has long since emerged to use therapeutic HIV vaccines that would help to retain the antiviral immune response suppressing viral replication and limiting the viral reservoir, as well as safeguarding in case of suboptimal adherence^9^. Initial success of such vaccines was limited, because of an insufficient quality or strength of the induced immune responses, incomplete coverage of HIV variability, and inappropriate immune activation^10^. More advanced multifaceted immunotherapeutic approaches were able to improve HIV-1-specific T-cell responses, reduce immune activation, and increase CD4 T-lymphocyte counts^10^. The latest developments including better antigen selection, more efficient vaccine delivery systems, combined interventions that stimulate the immune response and prevent new rounds of viral infection, as well as programming of T cell killers, are making functional HIV cure a feasible goal^11–13^. We proposed to complement the functional cure by vaccinating against primary drug-resistant mutations in reverse transcriptase, protease, integrase, and gp41, hypothesizing that such immunotherapy may create a “bottleneck” for viral evolution towards the resistant phenotype(s)^8,14–16^.

Implementation of this approach requires a multi-component vaccine. The most thoroughly explored HIV vaccines are multi-component DNA vaccines that have been tested in a series of preclinical and clinical trials^17–24^. A selection of these vaccines target complete or incomplete pol genes^23,25,26^ and gp41^27–29^. Plasmids encoding pol gene products were shown to be immunogenic in a series of preclinical and clinical trials^30–33^. However, a number of human and preclinical mouse trials revealed an impaired cellular immunogenicity of pol gene products, mainly, of the reverse transcriptase (RT)^34–38^. Mouse experiments with a multigene multiclade HIV vaccine revealed that only CD4+ T-cell responses against Pol exceeded the background level in the control group^39^. Furthermore, in some cases the addition of pol genes to multi-gene vaccines reduced the cellular responses to other components^38^ and interfered with the protection in a mouse model of HIV infection^37^. Altogether, these findings indicated the need to improve cellular immunogenicity of Pol.

RT is a key enzyme in viral replication. It is one of the major targets of ART and a primary focus of the attempts to achieve immune control over drug resistance. We conducted a series of preclinical trials aimed to induce an immune response to drug resistance-conferring mutations in RT, in order to include it into a multigene DNA vaccine against drug-resistant HIV-1^40–44^. However, RT encoded by viral genes was only weakly immunogenic^40,45^. We attempted to enhance its immunogenicity by redirecting RT to alternative pathways of antigen processing through fusion to various retargeting signals^35,36,42,46^. A significant improvement in immunogenic performance was achieved only in the case of RT retargeting to the lysosome^46^. We also tested whether RT can be made more immunogenic by expression optimization and artificial secretion, which we thought would help to overcome RT-induced oxidative stress, potentially toxic to the expressing cells^43^. However, artificial secretion conferred only minor changes to RT immunogenic performance. The cellular immune response induced by the secreted RT variant was still weak^43^. As the single approach-oriented tactics had failed, we performed the systematic optimization of all parameters defining gene immunogenicity, including gene design and the process of immunization. To promote MHC class I processing and the consequent induction of CD8+ T-lymphocyte-specific responses, we chose wild-type and drug-resistant RT variants (with enhanced proteasomal degradation)^47^, designed respective expression-optimized DNA immunogens, and optimized the entire immunization procedure. Specifcially, we tested different routes of DNA delivery, adjusted the electroporation parameters, applied different prime-boost regimens, and added an adjuvant that promotes a cellular immune response. Such systematic optimization was shown to considerably improve the immune response to DNA immunization, including its cellular component^48,49^. Indeed, we achieved significant enhancement of the anti-RT immune response. However, the response was of still of the Th2 type, involving primarily CD4+ T-cells and antibodies. The above procedures alone or in combination were unable to significantly enhance an anti-RT Th1 type immune response, indicating that the profile of the immune response is largely predetermined by the inherent properties of the encoded protein.

## Results

### Codon optimization results in efficient eukaryotic expression of the wild-type and drug-resistant RTs

In this study, we used RTs of the HIV-1 clade B HXB2 strain (RTwt) and the MN strain isolated from a patient with resistance to multiple NRTI^50^ (RT1.14; Supplementary Fig. S1). Viral genes were poorly expressed in the mammalian cells^50^. The level of eukaryotic expression correlates with the performance of DNA immunogens, and high expression levels commonly contribute to increased immunogenicity^51–54^. To increase the expression, we created synthetic coding sequences for RTwt and RT1.14 based on the codons frequently used in human cells, respective synthetic DNAs referred to as RTwt-opt and RT1.14-opt. Introduction of the Kozak sequence generated additional N-terminal Met-Gly residues (Supplementary Fig. S1). To make the RT genes safe as DNA vaccines, we inserted mutations abrogating the polymerase (D187N, D188N) and RNase H (E480Q) activities, these inactive RT variants referred to as RTwt-opt-in and RT1.14-opt-in (Supplementary Fig. S1). Effective inactivation was demonstrated in our *in vitro* studies^43,55–58^. Parental viral and newly generated synthetic RT genes were cloned into the eukaryotic expression vector pVax1 under the control of the immediate early CMV promotor.

**Figure 1:**
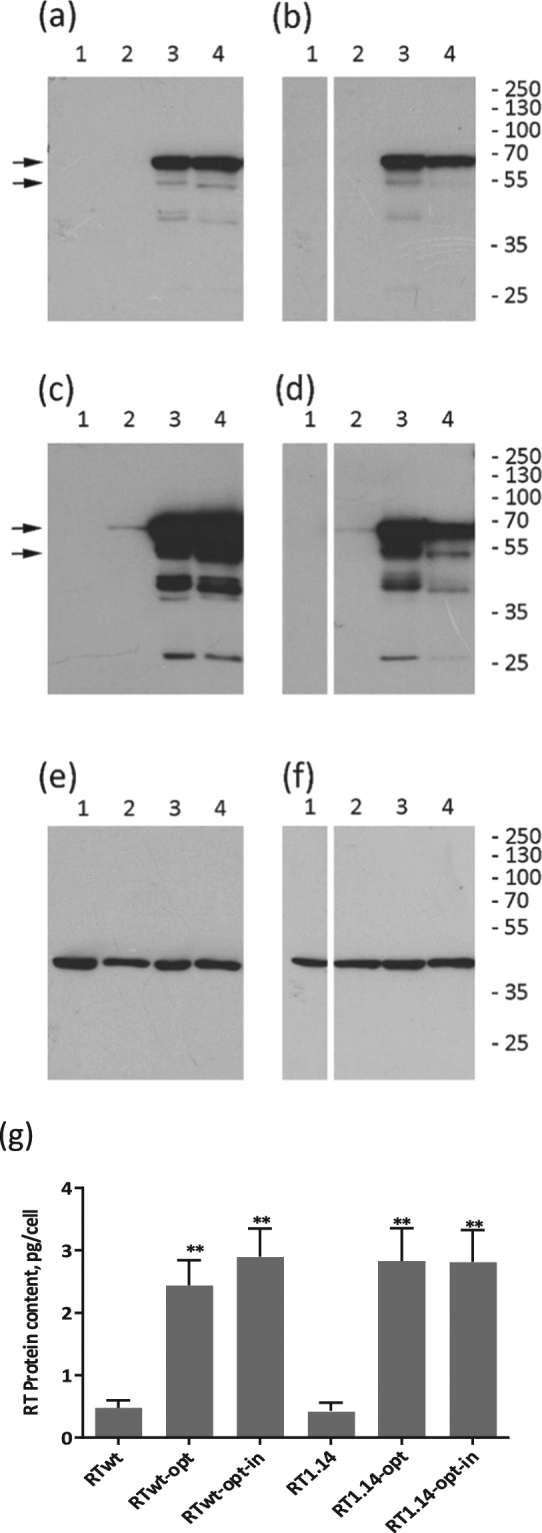
Expression of RT variants in eukaryotic cells. (**a**–**f**) Western blotting of the lysates of HeLa cells transfected with vector pVax1 (lane 1), and pVax-based plasmids expressing RTwt (lane 2 a,c,e), RTwt-opt (lane 3 a,c,e), RTwt-opt-in (lane 4 a,c,e), RT1.14 (lane 2 b,d,f), RT1.14-opt (lane 3 b,d,f), and RT1.14-opt-in (lane 4 b,d,f). Blots of RTwt and RT1.14 variants were processed in parallel. Blots were stained with rabbit polyclonal anti-RT antibodies^59^ (**a**–**d**) and then stripped and re-stained with monoclonal anti-actin antibodies. (**e**,**f**) Positions of the relevant molecular mass markers are given to the right in kDa. Arrows point at the p66 and p51 RT subunits. Panels (a,b) represent results of a 0.5 min exposure of X-ray film with a blot, while panels (c,d) represent results of a 10 min exposure. Full-length blots are presented in Supplementary Fig. 2. (**g**) The average amount of RT protein expressed per HeLa cell transfected with RT variant genes. **p < 0.01 (as compared to non-optimized RT variants) using the Mann–Whitney U-tests. The graph represents the results of three independent runs, each done in duplicate, and the error bars represent the SD.

All RT gene variants were tested for expression in mammalian cells. For this, HeLa cells were transfected with each of the plasmids, and after 48 hours cell lysates were collected and analysed by Western blotting. In all cases, we detected a protein with a molecular mass of 66 kDa stained by anti-RT antibodies^59^, and this staining was attributed to the p66 subunit of HIV-1 RT (Fig. 1a–d). Lysates of cells transfected with the synthetic RT genes contained a 51 kDa protein, also stained with anti-RT antibodies. This protein corresponded to the p51 subunit of RT formed due to processing of p66 by cellular proteases^60^. Quantification of the Western blots showed that all synthetic genes provided similar levels of expression of both RT subunits (p > 0.1); p51 expression reaching approximately 20% of p66 (Fig. 1a–d,g). This indicated similar processing of p66 variants by cellular proteases with generation of similar amounts of homodimers (p66/p66) and heterodimers (p66/p51) by all four enzyme variants (RTwt-opt, RTwt-opt-in, RT1.14-opt, and RT1.14-opt-in). Importantly, RT expression was not affected the mutations, either those conferring drug resistance or those abrogating RT activity (Fig. 1a–d,g). Lysates of cells transfected with viral RTwt and RT1.14 genes contained only p66 in amounts five times lower than in the lysates of cells transfected with the codon-optimized genes (Fig. 1a–d,g). In previous work, while studying the expression of RT chimeras targeted for secretion, we detected RT in the cell culture fluids of RT-expressing cells^43^. Here, too, we found both drug-resistant and wild-type RTs in the lysates and cell culture fluids in approximately equal amounts (Supplementary Fig. S3a and data not shown).

Next, we evaluated the polymerase activity of RT (done for RT1.14) contained in cell culture probes with a known percent of transfection. We related the activity to the amount of RT1.14 detected in the lysates and cell culture fluids by Western blotting, and calculated the activity per one expressing cell. This defined the ratio (in %, for convenience multiplied by 10^3^) of the enzyme that had retained polymerase activity. As an illustration, a specific RT activity equal to 41 meant that active enzyme constituted 0.0041% of the total amount of enzyme present in the fraction (Supplementary Fig. S3). Overexpression of RT1.14 from the synthetic RT1.14-opt gene led to a 10-fold decrease in the enzymatic activity (Supplementary Fig. S3). Thus, we found significantly reduced polymerase activity already after overexpression of the synthetic genes encoding enzymatically active RTs. Against this background, the introduction of the inactivating mutations gave little additional effect. Introduction of inactivating mutations gave no added value, supporting the notion that protein that is overexpressed in eukaryotic cells might lose its enzymatic activity due to aggregation^43^. Confirming this option, the aggregated protein was not secreted: the relative amount of the inactive RT1.14 in the cell lysate was 4–5-fold higher than in the secreted protein fraction (Supplementary Fig. S3).

Altogether, codon optimization resulted in efficient eukaryotic expression of both the wild-type and drug-resistant RTs. The amounts of both proteins reached up to 3 pg per expressing cell. The enzymes had negligible residual enzymatic activity, fulfilling the requirements for DNA vaccines to be applied in preclinical and clinical trials.

### Codon optimization of RT genes increased cellular and antibody responses to RT in DNA-immunized mice

A series of earlier studies demonstrated the effectiveness of intradermal/cutaneous DNA immunization for the induction of cellular immune responses^61–63^. Hence, BALB/c mice were immunized with RT genes by intradermal injections followed by electroporation. The intradermal DNA application was optimized as part of the RT DNA immunization regimen.

**Figure 2:**
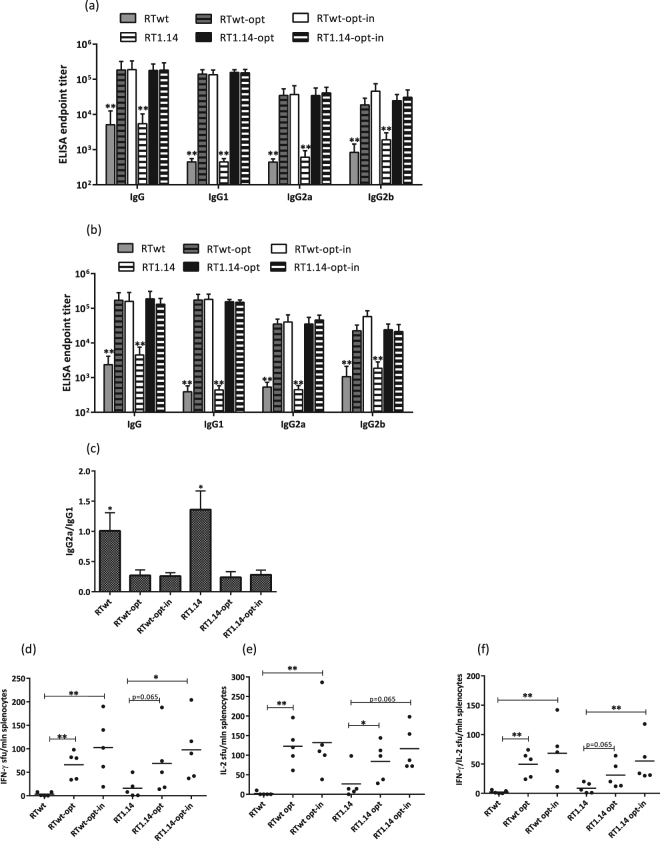
Immune responses following immunization with expression-optimized RT genes. BALB/c mice (n = 5 or 6) were immunized with two intradermal injections (29G needle) containing 20 µg of RTwt, RTwt-opt, RTwt-opt-in, RT1.14, RT1.14-opt, or RT1.14-opt-in encoding plasmids per mouse with subsequent electroporation by Dermavax (standard protocol) in two independent immunizations. At 21 days post-immunization mice were sacrificed, and sera and splenocytes were isolated for further immune tests. End-point average titers of anti-RT total IgG and IgG subtypes were detected using ELISA against recombinant RTwt and RT1.14 proteins with cut-offs set against the serum reactivity of control mice immunized with vector pVax 1. (**a**–**c**) ELISA with the identical RT protein variant received as a gene, i.e. recombinant RTwt protein for RTwt, RTwt-opt, and RTwt-opt-in immunized mice or RT1.14 for RT1.14, RT1.14-opt, and RT1.14-opt-in immunized mice. (**a**) ELISA with the homologous RT, i.e. recombinant RT1.14 protein for RTwt, RTwt-opt, and RTwt-opt-in or RTwt protein for RT1.14, RT1.14-opt, and RT1.14-opt-in immunized mice. (**b**) IgG2a/IgG1 ratio for antibody reactivity against RT variants matching RT used as DNA immunogen (**c**). In panels (a,b) the asterisk designates the difference between RTwt and RTwt-opt/RTwt-opt-in and between RT1.14 and RT1.14-opt/RT1.14-opt-in variants for all IgG subtypes. Murine splenocytes were stimulated *in vitro* with an RT-derived peptide representing the epitope of RT aa 528–543 (immunodominant T-cell epitope; Table 2) in the IFN-γ/IL-2 Fluorospot test (**d**–**f**). The average number of cells was registered as signal-forming units (sfu) per million splenocytes secreting IFN-γ (**d**) IL-2 (**e**) and IFN-γ/IL-2 (**f**) and the error bars represent the SD. *p < 0.05; **p < 0.01; tendency p-values are indicated on the graph. Statistical comparisons were performed using Kruskal-Wallis and Mann-Whitney tests.

In the first series of optimization experiments, mice received a single injection of RT gene variants. Three weeks later, mice were bled, sacrificed, and their spleens were collected. Serum samples were analysed for anti-RT antibodies by indirect ELISA on plates coated with corresponding recombinant RT variants (see “Materials and Methods”). In all cases, immunization with humanized genes led to a 10-fold increase in the levels of specific total anti-RT IgGs and of anti-RT IgG1, IgG2a, and IgG2b (p < 0.01; Fig. 2a). Sera of mice immunized with the RTwt and RT1.14 genes reacted equally strongly to the RTwt and RT1.14 proteins. The titer of antibodies against the RT variant used for immunization did not differ from the titer of antibodies against the homologous RT (p > 0.1; Fig. 2b). Codon optimization notably shifted the IgG2a/IgG1 balance towards stronger IgG1 production (Fig. 2a,c), which is characteristic of a Th2 type cellular response^64^.

We further assessed whether codon optimization had a similar positive impact on the cellular responses. Murine splenocytes were tested for their capacity to produce IFN-γ and IL-2 in response to stimulation with a peptide encompassing aa 528–543 of RT (RT528–543), which we and others have previously shown to represent a dominant T-cell epitope of RT in BALB/c mice^35,65^. Codon-optimized RT gene variants induced stronger cellular responses to RT528–543 in terms of single IFN-γ and IL-2 and dual IFN-γ/IL-2 production (Fig. 2d–f).

No difference was found between the cellular immune responses induced by RTwt and RT1.14 or by optimized active versus inactive RT gene variants (Fig. 2d–f). Thus, we demonstrated that, contrary to the earlier observations, inactivation of RT had no negative effect on its immunogenic performance^45^. In view of these findings, further optimization was carried out mainly using the inactivated expression-optimized RT gene variants RTwt-opt-in and RT1.14-opt-in.

### Repeated immunization with RT genes enhances both antibody and cellular immune responses

**Figure 3:**
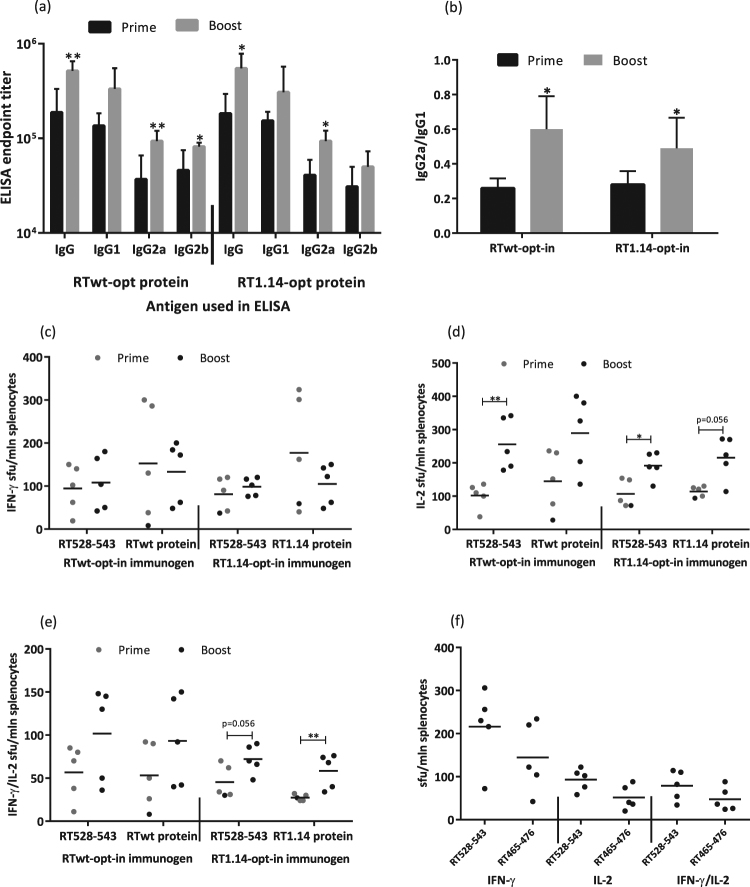
RT-specific antibody and cellular responses after repeated immunization with expression-optimized RT genes. BALB/c mice (n = 5 or 6) were immunized with two intradermal injections (29G needle) containing 40 µg of RTwt-opt-in or RT1.14-opt-in encoding plasmids per mouse, with subsequent electroporation by Dermavax (standard protocol). Immunizations were repeated 4 weeks later with 20 µg of RTwt-opt-in or RT1.14-opt-in encoding plasmids ((**a**–**e**) prime-boost regimen, “Boost” on the graph) or 5 days later with 20 µg of RTwt-opt-in encoding plasmid ((**f**) double prime). Two independent immunizations were performed. At 21 days after the second immunization, mice were sacrificed and sera and splenocytes were isolated and subjected to immune tests. Endpoint average titers of anti-RT total IgG and IgG subtypes (the error bars represent the SD) were detected using ELISA against recombinant RTwt and RT1.14 proteins with cut-offs set against serum reactivity of control mice immunized with vector pVax1 (**a**), and the IgG2a/IgG1 ratio was calculated for antibody reactivity against RT variants matching RT used as the DNA immunogen (**b**). Splenocytes were stimulated *in vitro* with RT-derived peptides representing epitopes of RT aa 528–543 (**c**–**f**) and aa 465–476 ((**f**) Table 2) or recombinant RTwt and RT1.14 proteins ((**c**–**e**) matching the immunogen) in IFN-γ/IL-2 Fluorospot tests (**c**–**f**). The average number of cells was registered as signal-forming units (sfu) per million splenocytes secreting IFN-γ (**c**,**f**) IL-2 (**d**,**f**) and IFN-γ/IL-2 (**e**,**f**) and the error bars represent the SD. IFN-γ/IL-2 production by splenocytes of mice receiving prime-boost immunization compared to single immunization ((**c**–**e**) single immunization from Fig. 2) or double prime immunization with the RTwt-opt-in gene (**f**). All assays were performed in duplicate. *p < 0.05; **p < 0.01; p-values on the graph in the interval 0.05 to 0.1 indicate tendencies. Statistical comparisons were performed using Kruskal–Wallis and Mann–Whitney tests.

After optimization of the gene immunogens, we proceeded to optimize the immunization regimen starting with different prime-boost protocols. BALB/c mice were primed and 4 weeks later boosted with the RTwt-opt-in or RT1.14-opt-in genes. Three weeks after the boost, mice were sacrificed, and their sera and spleens were collected and analysed for RT-specific humoral and cellular responses. The responses induced by prime-boost immunizations (referred to as “Boost” in the text and graphs) were compared with the responses induced by single immunizations (“Prime”). Prime-boost immunization enhanced the total anti-RT IgG and IgG2a responses induced by both immunogens, and also anti-RT IgG2b responses in RTwt-opt-inimmunized mice (Fig. 3a). The enhancement led to a shift in the IgG2a/IgG1 ratio from 0.2–0.3 registered after the prime to 0.5–0.6 registered after the boost. The prime-boost regimen had little effect on the RT-specific IFN-γ response in either the RTwt-opt-in or RT1.14-opt-in gene immunizations (Fig. 3c). Interestingly, however, it improved the RT-specific IL-2 and dual IFN-γ/IL-2 response specifically in the RT1.14-opt-in–immunized mice (Fig. 3d,e). The enhancement was significant in stimulations with the RT528–543 peptide and close to significant in stimulations with the recombinant RT1.14 (Fig. 3d,e). Thus, we were able to induce a slight shift in the response towards the Th1 type, but the response was still Th2 polarized.

Next, we tested the effect of the regimen dubbed “Double Prime”, according to which mice received RTwt-opt-in gene twice with a 5-day interval (other immunization parameters such as gene doses, type of injection, and regimen of electroporation were not changed). Three weeks after the second injection, mice were sacrificed and humoral and cellular responses were evaluated in sera and splenocytes. Results of the Double Prime were compared to those obtained after single and repeated prime-boost immunizations. Double Prime induced the same level of antibody response as single gene injections (data not shown). With respect to the cellular immune response, splenocytes of double-primed mice had a tendency for enhanced production of IFN-γ after stimulation with the RT528–543 peptide (p = 0.056) in comparison to IFN-γ in the single prime and prime-boost injection regimes, whereas IL-2 and dual IFN-γ/IL-2 responses were similar or even lower (Fig. 3c–e compared to Fig. 3f). Thus, Double Prime provided no major advantage over single injections or prime-boost regimens. Interestingly, however, in mice receiving double prime, we detected the recognition of RT aa 465–476, which contains a known human CD8+ T-cell epitope^66^ (Table 2 and Fig. 3f). This epitope was not recognized in Fluorospot tests performed after single RT gene injections or after prime-boost immunizations (data not shown). Thus, the Double Prime regimen, intended to provide more immunogen at the early stages of immune response development, had little effect on the potency of the response, but seemed to somewhat broaden the range of recognized T-cell epitopes. Finally, the classic prime-boost regimen was chosen as preferable because it induced both higher levels of specific antibodies and more potent IL-2 and dual IFN-γ/IL-2 production indicating the induction of a multifunctional T-cell response.

### Enhancement of the effector immune response in prime-boost DNA immunization as demonstrated by *in vivo* imaging

Based on the prime-boost regimen, we designed experiments to evaluate the effector capacity of the immune response. We have repeatedly shown in previous studies that the cellular immune response can be indirectly monitored *in vivo* by introducing the DNA immunogen together with a reporter gene, such as firefly luciferase (Luc), with further monitoring of the loss of signal emitted by the reporter^31,41,63^. We found that the photon flux from the sites of immunogen/reporter gene co-injections is inversely correlated to the cellular immune response against the immunogen^31,41,63^ and Petkov S. *et al*. (PLoS One [PONE-D-17-40839] [EMID: 2890cecde7221c73], accepted). Correlation of the loss of photon flux with the anti-Luc response was insignificant (data not shown) due to the low immunogenicity of Luc^31,41,63^. These observations established *in vivo* imaging as an animal-sparing method to evaluate the overall performance of DNA immunogens^31,41,63^.

To proceed with the *in vivo* evaluation of the effector potential of the immune response, we immunized mice with each of six RT-encoding plasmids mixed 1:1 (w/w) with the plasmid encoding Luc. The bioluminescence from the injection sites was monitored by *in vivo* imaging on days 1, 3, 9, 15, and 21 post immunization. A statistically significant decrease in the bioluminescence levels was observed in mice receiving Luc mixed with the expression-optimized drug-resistant and non-resistant RT if compared to the empty vector, or respective viral genes. The loss became dramatic by day 15 (p < 0.01 for RTwt versus RTwt-opt and for RTwt versus RTwt-opt-in; p < 0.01 for RT1.14 versus RT1.14-opt and for RT1.14 versus RT1.14-opt-in, Supplementary Fig. S4). When Luc DNA was administered with the expression-optimized RT genes, the bioluminescence signal decreased by 99% within 2 weeks and by 99.9% within 3 weeks after DNA delivery, whereas co-administration with the viral genes led to little or no reporter clearance (Supplementary Fig. S4). Loss of the luminescent signal coincided with the development of the cellular and antibody response against both RT forms (Figs 2 and 3). In agreement with the immune response data, there was no difference in the rate or extent of reporter clearance induced by the expression-optimized active and inactive RT gene variants (Supplementary Fig. 4) indicating that they induce an immune response with similar lytic/effector potential.

**Figure 4:**
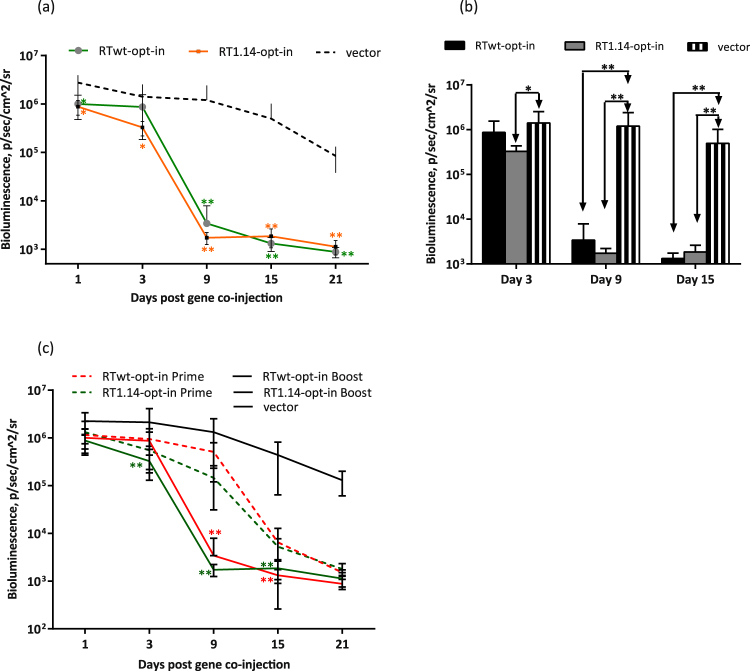
Effector immune response evaluated *in vivo* by “antigen challenge” in a prime-boost Luc/RT immunization of mice. BALB/c mice (n = 6) were immunized with two intradermal injections (29G needle) containing 40 µg RTwt-opt-in or RT1.14-opt-in encoding plasmids per mouse with subsequent electroporation by Dermavax (standard protocol) and 4 weeks later boosted with 20 µg of the same RT genes per mouse mixed 1:1 (w/w) with a Luc-encoding plasmid. Control mice received empty vector as a prime and a vector/Luc gene mixture as a boost. The emitted bioluminescence was monitored on days 1, 3, 9, 15, and 21 after boosting (**a**,**b**). Comparison of photon flux exhibited by mice primed with Luc/RT (Supplementary Fig. S4) or primed with RT and boosted with Luc/RT gene variants (**c**). Each curve (**a**,**c**) or bar (**b**) represents the average photon flux (photons/s/cm^2^/sr) from the injection area observed for the group of six mice (12 immunization sites), and the error bars represent the SD. The difference in photon flux between RT- and vector-immunized mice (**a**,**b**) and between prime and prime-boost groups of RT-immunized mice (**c**) is indicated by *p < 0.05 or **p < 0.01. In (**a**,**c**) the asterisk color matches the curve color. Statistical comparisons were performed using Mann–Whitney tests.

We used this technique to obtain *in vivo* evidence for the boosting of an anti-RT effector immune response. If functional, such a response should exterminate the immunogen/reporter co-expressing cells in mice boosted with RT gene variants faster than in mice immunized for the first time. To ascertain whether this was the case, mice were primed with the expression-optimized inactivated RT gene variants as described above, and 4 weeks later boosted with a 1:1 (w/w) mixture of Luc with the RT gene variant used for the priming. Loss of bioluminescence in mice primed with an RT gene and boosted with a mixture of this RT and Luc genes was compared to the loss of bioluminescence in mice receiving this mixture for the first time. A dramatic loss of bioluminescence was observed by day 9 after the boost, i.e. 1 week earlier than in mice immunized with RT/Luc mixture for the first time (Fig. 4a and Supplementary Fig. 4). This shift indicated the pre-existence of the RT-specific effector immune response induced by priming that was furthered by the booster injections. The loss of bioluminescence was similar for all expression-optimized RT gene variants (Fig. 4), indicating that the effector immune response they induced was of similar potency, i.e. it was unaffected by either the inactivation or drug-resistance conferring mutations.

### Penetrating delivery devices are preferential to non-penetrating devices in providing a more effective immune response to RT in DNA-immunized mice

The suboptimal elicitation of a cellular immune response by RT immunization precludes its promotion as an immunogen in primate trials. We attempted to improve RT’s cellular immunogenicity by optimizing the process of gene delivery. Appropriate DNA delivery improves the immunogenicity of DNA vaccines by enhancing the rate of *in vivo* transfection and by increasing antigen expression levels. The enhancement is also due to the indirect adjuvant effect(s) associated with trauma and subsequent inflammation at the sited of DNA delivery^49,67–69^. With this in mind, we optimized the delivery of two RT gene variants, RTwt-opt-in and RT1.14-opt-in, which we planned to test further in non-human primates.

The delivery regimen to be applied in order to achieve high levels of *in vivo* transfection was selected with the help of *in vivo* imaging. We injected mice with RT gene variants mixed with the Luc gene and followed Luc expression on days 1 and 3 post-delivery. At the experimental end point (day 21), we assessed the cellular immune response to both RT and Luc using IFN-γ/IL-2 Fluorospot. We expected on the best regimen to induce the highest Luc expression at the early time points (days 1, 3), indicating successful gene delivery, and a strong immune response to Luc and RT detected by *in vitro* cytokine secretion tests at the experimental end point.

We tested different injection modes, including insulin syringes with 29 G needles, microneedles (Micronjet 600, Nanopass Technologies, Israel), and a needle-free delivery device driven by CO_2_ (Biojector®2000, USA). Plasmid inoculations were followed by electroporation using a Dermavax device with multi-needle electrodes following the optimized regimen^70^. To reduce the invasiveness of electroporation, we immunized mice with a mixture of Luc/RT1.14opt-in genes with insulin syringes/29G needles and electroporated the injection sites using the non-penetrating plate-plate/flat (FL) electrodes (BTX, Japan). In the latter case, electroporation was carried out with a CUY21EditII electroporator (BEX Co. Ltd., Japan) because the Dermavax was not adapted for FL use. The electroporation regimen applied on the BEX machine mimicked that used with the Dermavax in all aspects except for the high voltage of the poration pulse (which is not achievable on the BEX device). Loss of bioluminescence indicating loss of Luc expression was expressed as the percentage of bioluminescence remaining in the inoculation sites in relation the highest level achieved at the site during the experiment, usually day 1 post immunization (Supplementary Fig. S5a).

On days 1 to 3, the level of bioluminescence at the sites of Luc/RT1.14-opt-in needle administration was 2–3 times higher than in Biojector deliveries (in both cases the sites were electroporated with the Dermavax; Supplementary Fig. S5). The microneedles provided greater Luc expression levels compared to both ordinary 29G needles and Biojector administrations (p < 0.05; Supplementary Fig. S5). The increase in Luc expression was observed not only in Luc/RT, but also also in Luc/vector-immunized mice (p < 0.05, Supplementary Fig. S5). Electroporation with non-invasive FL electrodes promoted lower levels of Luc expression than electroporation performed with the penetrating multi-needle electrodes (p < 0.05; Supplementary Fig. S5).

**Figure 5:**
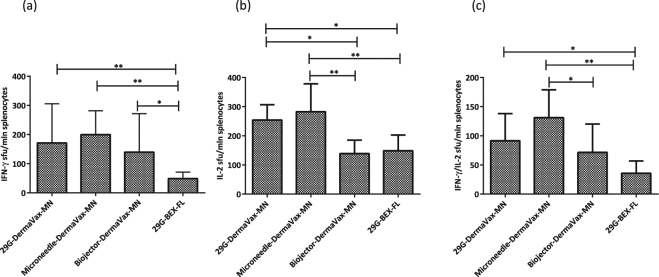
Cellular immune responses after RT gene delivery by intradermal injections with 29 G needles or microneedles with electroporation using penetrating electrodes. BALB/c mice (n = 6–8) were immunized with two intradermal injections with a mixture of Luc/RT1.14opt-in encoding plasmids (1:1 w/w, with a total of 2 × 20 µg DNA per mouse) using an insulin syringe with a 29G needle (29G-Dermavax-MN), microneedles (Micronjet 600, Nanopass) (Microneedle-Dermavax-MN), or a Biojector 2000 (Biojector-Dermavax-MN) and electroporated using Dermavax with multi-needle (MN) electrodes or injected with a mixture of Luc/RT1.14-opt-in encoding plasmids using an insulin syringe with a 29G needle and electroporated with the BEX machine and flat electrodes (29G-BEX-FL). At 21 days post immunization, mice were sacrificed and splenocytes were isolated and subjected to *in vitro* stimulation with peptide representing aa 528–543 of HIV-1 RT, and cytokine secretion was assessed by dual IFN-γ/IL-2 Fluorospot. The results are shown as the average number of cells, registered as signal-forming units (sfu) per million splenocytes secreting IFN-γ (**a**) IL-2 (**b**) and IFN-γ/IL-2 (**c**) and the error bars represent the SD. All assays were performed in duplicate. *p < 0.05; **p < 0.01. Statistical comparisons were performed using Kruskal–Wallis and Mann–Whitney tests.

Thus, needle injections followed by electroporation performed with multi-needle (MN) electrodes (29G-Dermavax-MN; Microneedle-Dermavax-MN) promoted the highest levels of bioluminescence/Luc expression on days 1 and 3 with a loss of 40–45% of the signal by day 9 and 98% of the signal by day 15 (Supplementary Fig. S5). Such kinetics of bioluminescence was earlier observed in the presence of a strong cellular immune response against the immunogen co-injected with the reporter^31,41,63^. Application of the regimens employing the Biojector or flat electrodes (Biojector-Dermavax-MN; 29G-BEX-FL) led to lower levels of bioluminescence by day 1 and the loss of signal already on day 3. The loss continued thereafter at a lower rate than the loss of bioluminescence in the first two groups (Supplementary Fig. S5), which indicated a weak immune response^31,41,63^. Indeed, at the end of the experiment splenocytes of mice immunized with RT1.14-opt-in using 29G ordinary needles/microneedles and electroporated with multi-needle electrodes demonstrated stronger IFN-γ, IL-2, and dual IFN-γ/IL-2 responses to stimulation with the RT528–543 peptide compared to mice immunized by Biojector or mice injected by 29G ordinary needles with subsequent electroporation using non-penetrating electrodes (Fig. 5).

### Optimization of electroporation

After optimization of the immunogens, the immunization scheme, and the delivery vehicles, we proceeded to optimize the process of electroporation because this can greatly improve the performance of DNA vaccines^48,71^. The efficacy of electroporation is determined by the combination of several parameters, such as voltage, duration of the pulses, pulse polarity, and configuration of the electrodes. These parameters were tested in electroporations performed using a CUY21EditII device (BEX Ltd., Japan; referred to here and below as “BEX”), which allowed the use of different configurations of electrodes. In a preliminary experiment, we demonstrated that mice receiving single injections of the RTwt-opt-in genes followed by electroporation delivered by BEX and by the already tested Dermavax (Cellectis) mounted similar IFN-γ, IL-2, and dual IFN-γ/IL-2 responses to a “reporter” epitope of HIV-1 RT (p > 0.1; Supplementary Fig. S6). The latter demonstrated that the BEX and Dermavax electroporators performed with a similar efficacy.

#### Voltage

**Figure 6:**
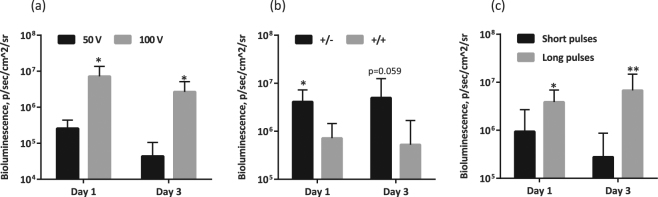
Evaluation of the driving pulse voltage (**a**) polarity (**b**) and duration (**c**) effects on the delivery/expression of the luciferase reporter gene. BALB/c mice (n = 4–8) were intradermally injected with 20 µg pVaxLuc (per site) into two sites, and injections were followed by electroporation with a BEX machine equipped with multi-needle electrodes (CUY691-5, BEX Ltd). Electroporation was initiated with a poration pulse of 400 V of 0.05 ms followed by a train of eight 50 V (**a**–**c**) or 100 V (**a**) pulses of the same (+/+; **a**–**c**) or opposing/alternating polarity (+/−; **b**,**c**) administered for 10 ms (Short pulses; **a**–**c**) or 50 ms (Long pulses; **b**,**c**) with 20 ms (Short pulses) or 950 ms (Long pulses) intervals. Bioluminescence was registered by *in vivo* imaging on day 1 and 3 after the injections. Data represent the average photon flux from all injections sites in the group (photons/s/cm^2^/sr), and the error bars represent the SD. All assays were performed in duplicate. *p < 0.05, **p < 0.01; p-values in the interval 0.05 to 0.1 indicate tendencies. Statistical comparisons were performed using Mann–Whitney tests.

A review of the methodology of DNA vaccination for small laboratory animals (such as mice and rabbits) recommends the use of electric pulses of 50 V to 100 V^48^. However, Lin *et al*. described the advantages of low-voltage electroporation (<60 V) for both immunogen expression and the induction of a cellular immune response^72^. To clear this inconsistency, we performed a series of experiments aimed to define the optimal regimen to be applied in mice. Most of the optimizations were done using standard MN electrodes in order to relate the results of the optimization to the levels of immunogenicity achieved using the electroporation regimen optimized for Dermavax^70^. First, we compared the efficacy of reporter gene delivery at 50 V versus 100 V. Unlike the findings of Lin *et al*.^72^, a train of 100 V pulses gave significantly higher levels of luciferase expression than a train of pulses at 50 V (Fig. 6a). Pulses of lower voltage (15 V and 30 V) were even less efficient with all the electrodes that we tested (Supplementary Fig. S7 and data not shown).

#### Pulse polarity

Alternating/opposing polarity (+/−) might support higher efficacy of *in vivo* transfection than unipolar pulses (+/+)^73,74^. Confirming these observations, test deliveries of the Luc reporter gene by electroporation with alternating pulses promoted significantly higher levels of bioluminescence than unipolar pulses (p < 0.05 for days 1 and 3 post administration; Fig. 6b).

#### Pulse duration

Electroporation manuals provided by the manufacturer (BEX) recommended the use of comparatively long 50 ms driving pulses. Indeed, at 50 V the application of the shorter pulses resulted in a lower level of bioluminescence (Fig. 6c). Attempts to perform the electroporation for 50 ms at 100 V, which was the optimal voltage for expression (Fig. 6a), were terminated due to the traumatic character of such long pulses for the mice. The limited results we obtained in the pilot 100 V tests made on four animals (two mice with two injection sites per regimen) did not show any difference in the reporter expression between the long- and short-pulse application (data not shown). Accordingly, the 100 V electroporation regimen was chosen with a less traumatic train of short 10 ms driving pulses. Such pulses are also easier to administer because they imply a shorter overall duration of the procedure, which is important specifically in case of large-scale DNA vaccine trials in mice.

#### Electrodes

When optimizing the regimen of RT gene immunization, we found that electroporation with penetrating MN electrodes was preferable to electroporation performed with non-penetrating electrodes, both in terms of gene expression and in terms of the induced cellular immune response (Supplementary Fig. S6, Figs 5 and 6). We concluded the electrode comparison with a head-to-head testing of the MN electrode with other penetrating electrodes, namely the fork-plate and two-needle types (Supplementary Fig. S7). Penetrating fork-plate electrodes were superior to the MN electrodes in terms of the ability to promote high levels of reporter expression by day 3. Two-needle electrodes were inferior to both fork-plate and MN electrodes, which argues against their further use in DNA immunization (Supplementary Fig. S7).

In summary, we established an optimal EP regimen constituted by short 10 ms 100 V pulses of electric current of opposed polarity delivered with penetrating electrodes that provided sufficient levels of early gene expression to support an efficient cellular immune response to a DNA immunogen. CUY21EditII (BEX) not only registered voltage and skin resistance at the site of the electrode’s application, but also imposed tight control of the electric current (execution is disabled if the current exceeds the limit). This measure protects against EP-induced injuries, making this device preferable for further preclinical development as well as clinical applications.

### The use of cyclic di-GMP adjuvant in RT DNA immunization increases the early cellular response

One of the strategies to shift the immune response towards the Th1 type is the use of molecular adjuvants. For this study, we selected cyclic di-GMP (c-di-GMP), a mucosal adjuvant known to enhance the cellular response by boosting the innate immunity and to skew the response towards the Th1 type^75–78^.

**Figure 7:**
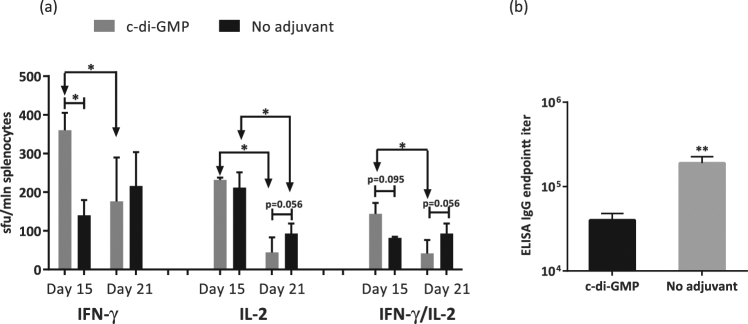
The effect of c-di-GMP adjuvant on the specific IFN-γ/IL-2 immune response in mice immunized with RT genes. BALB/c mice (n = 5) were immunized on days 1 and 5 with two intradermal injections of 20 µg RTwt-opt-in encoding plasmid per mouse (standard protocol with EP) followed by intraperitoneal injections of 1 nmol c-di-GMP (“c-di-GMP” group) or PBS (“No adjuvant” group), and control mice were similarly treated with the empty vector pVax1. On day 15, mice were bled and their PBMCs were collected and stimulated with peptide representing the immunodominant epitope of RT (RT528–543; Table 2). Cytokine production in response to stimulation was assessed by IFN-γ/IL-2 Fluorospot (**a**) On day 21, mice were sacrificed and their splenocytes were collected and subjected to the same IFN-γ/IL-2 test. (**a**) The results represent the average number of cells, registered as signal-forming units (sfu) per million splenocytes secreting IFN-γ, IL-2, and IFN-γ/IL-2, and the error bars represent the SD. (**b**) Endpoint (day 21) titers of anti-RT total IgG detected in ELISAs against recombinant RTwt protein with cut-offs set against the serum reactivity of control mice immunized with the vector pVax1. All assays were performed in duplicate. *p < 0.05; p-values in the interval 0.05 to 0.1 indicate tendencies. Statistical comparisons were performed using Mann–Whitney tests.

We immunized BALB/c mice with RTwt-opt-in using the double prime regimen in the presence of c-di-GMP delivered as recommended^79^. On day 15 after immunization, mice were bled, and peripheral blood mononuclear cells (PBMCs) were purified, stimulated with the RT528–543 peptide, and analysed for IFN-γ/IL-2 production by the Fluorospot test. Mice that received c-di-GMP presented a two-fold greater number of cells responding to RT-specific stimulation by secretion of IFN-γ and dual IFN-γ/IL-2 than mice receiving a non-adjuvanted immunization (Fig. 7a). The difference in IL-2 response between the two groups was insignificant (Fig. 7a). At the experimental end point on day 21, we analysed the responses of murine splenocytes. Cellular responses in mice receiving the RT-DNA gene in the presence of c-di-GMP did not differ from those in mice receiving the RT-DNA gene without c-di-GMP, although there was a tendency for lower IL-2 and dual IFN-γ/IL-2 responses in those not receiving the adjuvant (Fig. 7a). In the adjuvanted mice, the magnitude of the IFN-γ and IFN-γ/IL-2 response exhibited at week 3 decreased in comparison to the response by day 15 (p < 0.05), which was not the case for mice receiving non-adjuvanted immunization (Fig. 7a). We also analysed the titers of total anti-RT IgG antibodies in c-di-GMP adjuvanted mice. The final titer was 40,000 ± 8,000, which was almost five times lower than that in the non-adjuvanted animals (190,000 ± 36,000; p < 0.01; Fig. 7b). Thus, the application of c-di-GMP shifted the response towards Th1 by lowering the antibody response, but without a sustained improvement of the cellular responses.

### Drug-resistant and wild-type RT genes differ in the response they induce against the regions of drug-resistance mutations

**Figure 8:**
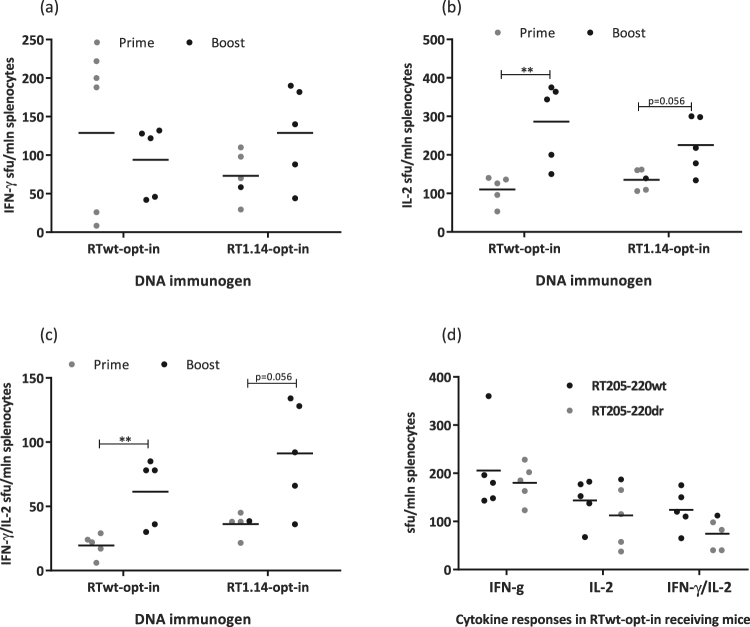
Cross-recognition of wt/dr proteins and peptides in RT gene-immunized mice. Mice were immunized as described in Figs 2 and 3. RT1.14 and RTwt proteins were used to stimulate splenocytes of mice immunized (either in the prime or prime-boost regimen) with RTwt-opt-in and RT1.14-opt-in encoding plasmids, respectively (**a**–**c**). RT205–220 wt/dr peptides (Table 1; **d**) were used to stimulate splenocytes of mice immunized (in the prime regimen) with the RTwt-opt-in encoding plasmid (**d**). Results of the Flurospot test detecting IFN-γ, IL-2, and dual IFN-γ/IL-2 production are presented as the average number of cells, registered as signal-forming units (sfu) per million splenocytes secreting cytokines, and the error bars represent the SD. All assays were performed in duplicate. **p < 0.01; p-values in the interval 0.05 to 0.1 indicate tendencies; p > 0.1 for comparison of responses to RT205–220 wt and RT205–220 dr (**d**). Statistical comparisons were performed using Mann-Whitney tests.

The goal of RT gene immunization is to target the immune response to drug-resistant HIV-1. We therefore inquired whether, after optimal immunization with RT, we can detect an immune response to peptides encompassing the regions of drug resistance (DR) mutations. To answer this question, we immunized mice with expression-optimized wild-type (RTwt-opt-in; n = 5) and drug resistant (RT1.14-opt-in; n = 5) RT genes by 29 G needle injections followed by optimal eleectroporation. At the experimental end point, murine splenocytes were stimulated with the recombinant wild-type and drug-resistant RT proteins and individual peptides covering the regions of DR mutations (Table 1). Proteins matching the immunogens were recognized already after the prime. A second injection tended to boost IL-2 and IFN-γ/IL-2 responses in RT1.14-immunized mice (Fig. 3d,e). The initial level of cross-recognition of the mismatched RT proteins was low (Fig. 8a–c). Cross-recognition, especially IL-2 and dual IFN-γ/IL-2 secretion in response to stimulation with the mismatched recombinant RT, improved noticeably after the boost (Fig. 8b,c). Also cross-recognized were the peptides with and without DR mutations RT205–220 wt and RT205–220 dr (Fig. 8d and Table 1) encompassing an immunodominant T-cell epitope recognized in mice^80^.

**Figure 9:**
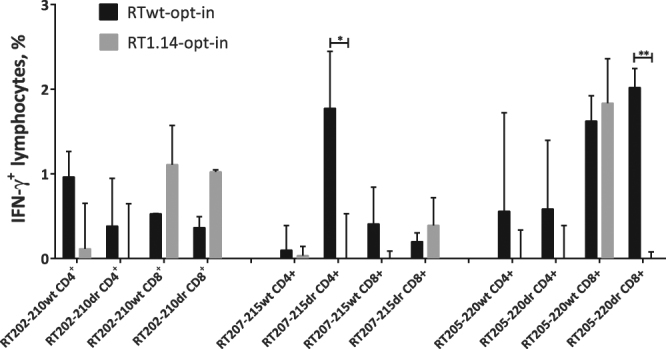
CD4+ and CD8+ epitope mapping in the 202–220 RT region as assessed by multiparametric FACS. Epitope mapping of CD4+ and CD8+ T-cell recognition by RTwt-opt-in and RT1.14-opt-in gene-immunized mice (pool of 5 mice) of aa 202–220 of RT with and without drug-resistance mutations (Table 1) assessed by multiparametric FACS with staining for CD4+, CD8+, and IFN-γ. Immune assays were performed following standard protocols on the murine splenocytes isolated 21 days after immunization with the RT gene. Assays were performed in duplicate. For the description of the immunization experiment, see the legend to Fig. 2 and “Materials and methods”. Responses are presented as the percentage of the reactive CD4+ and CD8+ cells producing IFN-γ, and error bars show the SD. *p < 0.05, **p < 0.01. Statistical comparisons were performed using paired t-tests.

To map the immune recognition of DR mutations in detail, murine splenocytes were stimulated with a panel of peptides covering the regions of the mutations. The corresponding cellular responses were assessed by IFN-γ/IL-2 Fluorospot and multi-parametric flow cytometry. A peptide was considered to be recognized if in immunized mice it induced >50 IFN-γ-, IL-2-, and/or IFN-γ/IL-2-producing T-cells per million splenocytes. The data are illustrated in Figs 8 and 9 and Table 1 that contain an overview of the results of IFN-γ/IL-2-Fluorospot tests performed after a series of single and double (double prime and prime-boost) immunizations with the RTwt-opt-in and RT1.14-opt-in genes.

We detected prominent reactivity to the immunodominant mouse epitope at aa 199–220^80^ (Table 1; Figs 8 and 9). Immunization with the RTwt-opt-in gene induced a cellular response capable of recognizing RT aa 202–220 both with and without DR mutations (Table 1 and Fig. 8d). At the same time, immunization with the drug-resistant RT1.14-opt-in gene induced no response against the epitopes contained within aa 199–220 as measured by IFN-γ/IL-2 Fluorospot (Table 1). Low/no reactivity indicated that DR mutations at aa 202, 207, 214, and 215 alone or in combination disrupted the immunodominant T-cell epitope(s) contained in this region making it non-immunogenic. No specific IFN-γ, IL-2, or dual IFN-γ/IL-2 secretion of splenocytes of either RTwt-opt-in or RT1.14-opt-in immunized mice was detected in response to stimulation with RT 61–69 wt/dr or with the pools of peptides covering aa 63–82 with or without DR mutations (data not shown).

**Table 1:** Immune recognition by RTwt and RT1.14 gene-immunized mice of the peptides encompassing regions of drug resistance conferring mutations detected by IFN-γ/IL-2 Fluorospot.

Peptide used to stimulate murine splenocytes*	Cytokine responses raised in mice after immunization with RT genes					
	RTwt-opt-in			RT1.14-opt-in		
	IFN-γ	IL-2	IFN-γ/IL-2	IFN-γ	IL-2	IFN-γ/IL-2
RT 199–216 wt	>100	50–100	50–100	<50	<50	<50
RT 199–216 dr	>100	>100	>100	<50	<50	<50
RT 202–210 wt	**<50**	<50	<50	**<50**	<50	<50
RT 202–210 dr	**<50**	<50	<50	**<50**	<50	<50
RT 205–220 wt	>100	>100	>100	**<50**	<50	<50
RT 205–220 dr	>100	>100	>100	<50	<50	<50
RT 207–215 wt	50–100	<50	<50	<50	<50	<50
RT 207–215 dr	50–100	50–100	<50	**<50**	<50	<50

Each entry represents the results of two or more IFN-γ/IL-2 Fluorospot tests performed after single or repeated (double-prime and prime-boost) RT gene immunizations: >100 – more than 100, 50–100–50 to 100, <50 - less than 50 spot-forming cells per million stimulated lymphocytes (splenocytes). *Peptide sequences are given in Table 1. Results of Fluorospot tests that are discordant with the results of the multi-parametric FACS assays (Fig. 9) are in bold type. For the description of the immunization experiment, see “Materials and methods” and legends to Figs 2 and 3.

To characterize this phenomenon further, we stimulated splenocytes of RTwt-opt-in and RT1.14-opt-in gene-immunized mice with peptides representing aa 202–210, 205–220, and 207–215 of RT with or without DR mutations. *In vitro* recognition of the peptides was analysed by multiparametric FACS. We detected cellular reactivity to peptides RT207–215 and RT202–210 with and without DR mutations (Fig. 9) that had been detected as weak (aa 207–215) or had been missed (aa 202–210) by IFN-γ/IL-2 Fluorospot (Table 1, see the numbers in bold type). IFN-γ secretion by CD4+ T-cells of RT gene-immunized mice in response to stimulation with peptides RT202–210, RT205–220, and RT207–215 detected by FACS (Fig. 9) indicated the presence of at least two murine CD4+ T-cell epitopes in this region, one localized within aa 202–210 and the other within aa 205–220. The CD4+ T-cell response against RT202–210 (wt and dr), RT207–215 (dr), and RT205–220 (wt and dr) was induced only in RTwt-opt-in and not in RT1.14-opt-in–immunized mice (Fig. 9). At the same time, both RT gene variants induced a CD8+ T-cell response to mutated and non-mutated variants of RT202–210 (Fig. 9). Also, both RT genes induced a strong CD8+ T-cell response against the wild-type (but not DR) variant of RT205–220. The DR variant of RT205–220 was recognized exclusively by mice immunized with RTwt-opt-in (Fig. 9). These observations attributed the cellular reactivities missed by IFN-γ/IL-2 Fluorospot to CD8+ T cells (Table 1 and Fig. 9).

Thus, we found that only the wild-type RT gene was capable of inducing an immune response to CD4+ T-cell epitope(s) located at aa 202 to 220 of RT that bears a cluster of drug-resistance conferring mutations. Using the drug-resistant RT1.14 derived from the virus as DNA immunogen resulted in a loss of immune recognition of a promiscuous CD4+ T-cell epitopic cluster localized at aa 202–220, as well as the loss of recognition of a CD8+ epitope localized at aa 205–220.

## Discussion

The ability of the host to successfully combat a pathogen is determined by the strength and phenotype of the pathogen-specific immune response. Classical Th phenotypes are Th1, associated with the cellular, and Th2, associated with the humoral immune responses^81^. DNA vaccines are known to induce a Th1 response. The potency of immune response, specifically in large species, has long been their weak point, that has only lately been overcome by optimization of gene expression and gene delivery techniques^48^. A Th1-biased immune response is thought to be due to the intracellular expression of the immunogen (also by cells of the immune system) and its prefential presentation by MHC class I molecules^82,83^. However, this Th1 bias does not preclude the induction of strong antibody responses^84–86^ enabling to use DNA immunization as a platform for generation of monoclonal antibodies^87^. This demonstrates that despite certain success in potentiating DNA vaccines, we still lack a complete understanding of the determinants of their immunogenicity. A closer look reveals that the strength of the immune response and its Th polarization is predetermined by multiple factors including the nature of the DNA immunogen (its toxicity, stability, routes of processing), the site of immunization/antigen presentation, the nature of the uptaking antigen-presenting cells (APCs) and their migration patterns. A good deal of these parameters is predefined by the vaccine delivery technique^88–90^.

In this study, we aimed to enhance the Th1 arm of the immune response against HIV-1 RT, in-going into the prototype multigene vaccine against drug-resistant HIV^14^ (see^15^ for review). Our earlier studies have shown that RT DNA is insufficiently immunogenic^40,45^. Expression optimization complemented by recent developments in DNA delivery/immunization techniques can significantly improve the immunogenicity of DNA vaccines^71,91,92^, and we reasoned that a systematic application of these techniques would help to increase the magnitude and breadth of the immune response induced by RT genes, opening possibility of their vaccine application(s). These experiments would also help to define the effect of systematic optimization on the terminal outcome of Th polarization.

We started with gene optimization using drug-resistant and non-resistant variants of RT of HIV-1 clade B strains. Parental RT variants included wild-type RT of HIV-1 HXB2 (RTwt) and RT with mutations derived from a drug-resistant HIV-1 MN strain (RT1.14)^40^ conferring resistance to multiple NRTI. Compared to the human genome, HIV-1 has a biased nucleotide composition. It is not fit to promote a high level of expression of the encoded proteins and is apparently responsible for the virus’s immunomodulatory and possibly also pathogenic properties^53,93,94^. To change this, we used a standard technique of codon optimization for mammalian cells that ensures the level of immunogen expression sufficient for the induction of a potent immune response^95–98^. For both the RTwt and RT1.14 genes, codon optimization increased the level of eukaryotic expression by five fold. Codon-optimized genes directed the synthesis of the p66 subunit of RT, which was processed in the expressing cells to p51 subunit that constituted up to 20% of the recombinant protein. Processing allowed the formation of RT heterodimers presenting the enzymes to the immune system in the conformations and forms seen in HIV-1 infection. We made the RT-based immunogens safe by introducing the inactivation mutations abrogating hazardous (polymerase) as well as potentially hazardous (RNAse H) enzymatic activities^55–58^ (the polymerase activity was reduced to <0.005% of the initial).

The resulting gene immunogens were assessed for immunogenicity in mice. The antibody response induced in mice by all of the codon-optimized RT genes was an order of magnitude higher than the response induced by the wild-type viral genes: anti-RT antibody titer reached 2 × 10^5^ after single immunizations and 5 × 10^5^ after booster immunizations. The cellular response to RT, specifically IFN-γ/IL-2 production, also increased 2–3 fold to 200 IFN-γ and IL-2-producing cells per million splenocytes. Thus, codon optimization enhanced the performance of RT in DNA immunization. Unlike our early experiments with viral RT genes, synthetic genes encoding inactivated RT were as immunogenic as genes encoding active enzymes, which we attributed to high expression levels of all RT variants^40,50^. Mice immunized with the expression-optimized RT genes demonstrated strong Th2 polarization of the immune response as manifested by low levels of IFN-γ production and a low anti-RT IgG2a/IgG1 ratio^64^. Thus, on the overall, the expression-optimized RT genes acted as Th2 type immunogens, i.e. in the same way as the parental RT genes.

**Table 2:** List of peptides used in T-cell *in vitro* tests.

Abbreviated name	Amino acid sequence in the regions of DR mutations	DR mutations (if applicable)	T-cell epitope (if applicable)

RT_wt_opt-in	TPVFAIKKKDSTKWRKLVDFRELNKRTQ		
RT_1.14_opt-in	TPVFVIKKKNNTGWRKLIDLRELNKRTQ		
**PEPTIDES**			
RT 61–69 wt	---FAIKKKDST----------------	A62V, D67N, S68N	N/A
RT 61–69 dr	---FAIKKKNST----------------	A62V, D67N, S68N	N/A
RT 63–72 wt*	-----IKKKDSTKWR-------------	D67N, S68N, K70G	N/A
RT 63–72 dr*	-----IKKKNNTGWR-------------	D67N, S68N, K70G	N/A
RT 66–76 wt*	--------KDSTKWRKLVD---------	D67N, S68N, K70G, V75I	N/A
RT 66–76 dr*	--------KNNTGWRKLVD---------	D67N, S68N, K70G, V75I	N/A
RT 74–82	----------------LVDFRELNK---	V75I, F77L	N/A

RT_wt_opt-in	RTKIEELRQHLLRWGLTTPDKKHQKEPPFLWMGYEL		
RT_1.14_opt-in	RTKVEELREHLLRWGFYTPDQKHQKEPPFLWMGYEL		
**PEPTIDES**			
RT 199–216 wt	RTKIEELRTHLLRWGLTT------------------	I202V, Q207E, L214F, T215Y	N/A
RT 199–216 dr	RTKVEELREHLLRWGFY-------------------	I202V, Q207E, L214F, T215Y	N/A
RT 202–210 wt	---IEELRQHLL------------------------	I202V, Q207E	N/A
RT 202–210 dr	---VEELREHLL------------------------	202V, Q207E	N/A
RT 205–220 wt	------LRQHLLRWGLTTPDQK--------------	Q207E, L214F, T215Y, K219Q	N/A
RT 205–220 dr	------LREHLLRWGFYTPDQK--------------	Q207E, L214F, T215Y, K219Q	N/A
RT 207–215 wt	--------QHLLRWGLT-------------------	Q207E, L214F, T215Y	N/A
RT 207–215 dr	--------EHLLRWGFY-------------------	Q207E, L214F, T215Y	N/A
**Peptides covering regions not subjected to DR mutations**			
RT 465–476	KVVPLTNTTNQK	N/A	CD8+ T-cells^(a)^
RT 514–528	ESELVNQIIEQLIKK	N/A	CD8+ T-cells^(b)^
RT 528–543	KEKVYLAWVPAHKGIG	N/A	CD4+ T-cells^(c)^

*RT 63–72 wt/RT 66–76 wt and RT 63–72 dr/RT 66–76 dr were used in pools called RT 63–76 wt and RT 63–76 dr, respectively.

References: (a) -^43^; (b) -^130^; (c) –^65^.

The RT-specific cellular responses, crucial for the control of HIV replication^99,100^, remained comparatively low, prompting further optimization. Specifically, we tested whether we could increase the T-cell response by increasing the gene dose (beyond a total of 40 µg which can be administered in one intradermal immunization). For this, we delivered RT-DNA by repeated injections administered over a short time interval (Double Prime). This strategy led to an increase in the number of targeted CD8+ T-cell epitopes, specifically the induction of a response to a CTL epitope of RT at aa 465–476, and tended to increase the overall IFN-γ production. However, IL-2 and IFN-γ/IL-2 levels remained the same or became lower (with time) in comparison to those achieved after a single immunization. A prime-boost regimen in which the RT gene was injected twice with a month’s interval was more successful. With this we were able to boost the production of IL-2 and IFN-γ/IL-2, i.e. the induction of a multifunctional T-cell response. Altogether, by optimizing the RT gene and the immunization regimen we were able to significantly elevate cellular/IFN-γ responses. Flow cytometry analysis attributed the majority of these responses to CD4+ T-cells (Fig. 9). In all three regimens tested, synthetic RT genes retained their Th2 profile with a largely unchanged IgG2a/IgG1 ratio. These results resemble those of an earlier study on DNA immunization with the expression of optimized HIV-1 Gag genes that induced a strong Th2-biased antibody response and no cytotoxic T-lymphocytes, the Th2 polarization attributed to a large amount of immunogen available to the immune system^53^.

Insufficient cellular responses promoted further efforts to optimize the process of immunization. The performance of DNA vaccines can be significantly improved by better gene delivery with more cells (including immune cells) presenting immunogen to the immune system of the host^101,102^. We tested different delivery vehicles, comparing an ordinary 29G-needle insulin syringe, microneedles, and a CO_2_-driven Biojector. Despite success in clinical trials^26,103^, in our hands the Biojector appeared to be inferior to both ordinary 29G needle and microneedle modes of DNA delivery. The latter two performed well in both the efficacy of gene delivery/expression and the induction of immune response, advocating for the application of the penetrating delivery devices. Although microneedles perform somewhat better, their use may be limited by their high cost.

We also optimized the next step in gene delivery, the electroporation, which is the gold standard for the delivery of macromolecules into the skin. The electroporation acts at two levels, by enhancing penetration of DNA into the cells and by creating an adjuvant effect linked to the local damage of the electroporated tissues with concomitant inflammation^104,105^. Traumatization might be very helpful in the improvement of immunogenic performance, but has to be acceptable and should not cause a strong tissue damage, in order not to hamper the immune response^67,106–108^. In our previous studies, we employed Dermavax EP (Cellectis) and MN electrodes^70^. Here, to optimize the electroporation regimen, we employed a CUY21EditII electroporator (BEX), which provides tight control over the electric current, with a set of penetrating and non-penetrating electrodes. Short driving pulses of 100 V of opposed polarity were selected as the least traumatic and superior to the other regimens both in terms of the initial level of expression of DNA immunogen and in terms of the potency of the immune response at the experimental end-point. Longer pulses were excluded for ethical reasons due to their traumatic nature. An extensive trauma might also have adverse effects on the immune response because of permanent permeabilization of cell membranes and the initiation of cell death^104^. We recently obtained *in vivo* confirmation of the high efficacy of the immune response induced by this regimen. DNA immunization with Luc with electroporation performed as short 100 V pulses of opposed polarity protected mice from the challenge with Luc-expressing syngenic tumor cells, whereas the same regimen at 50 V was ineffective^109^. Finally, we tested whether the responses can be enhanced by the application of different electrodes. Unfortunately, the attractive non-penetrating flat electrodes were not sufficiently effective either in driving *in vivo* transfection/gene expression or in ensuring an effective immune response at the experimental end-point. Multi-needle and fork-plate electrodes demonstrated similar efficacy, while the two-needle electrodes were inferior to both. Thus, more “invasive” penetrating electrodes might be recommended as effective in terms of both gene delivery and the induction of an immune response.

Finally, we attempted to shift the anti-RT immune response to the Th1 type response by using molecular adjuvants, specifically, the mucosal adjuvant cyclic di-GMP (c-di-GMP)^75,76,78,110^. By using c-di-GMP, we intended to stimulate the detection of double-stranded DNA by cytosolic DNA sensors, with the induction of subsequent downstream immune signaling^111^. Indeed, the use of this adjuvant diminished anti-RT antibody responses by five fold indicating a diminished Th2 polarization. It also served to induce the early development of an IFN-γ and IFN-γ/IL-2 response. Unfortunately, the effect of c-di-GMP on the cellular responses was not long lasting. Altogether, the extensive optimization of the immunogen and of the immunization process in a prime-boost regimen with the application of 29G needles/microneedles for gene delivery and the optimal controlled electroporation with penetrating electrodes increased the magnitude of the antibody response much more than the magnitude of the cellular response, with HIV-1 RT still performing as a Th2-polarized immunogen.

In culmination, we performed an *in vivo* evaluation of the effector immune response induced in mice after the optimal RT gene immunization. We have previously shown that when a DNA immunogen is co-delivered with a reporter gene, the immune response against the co-delivered DNA immunogen correlates with the loss of reporter expression^31,41,63^. The reporter expression can then be used as a surrogate marker of the capacity of the immune response to exterminate the expressing cells. Based on this, we devised a surrogate “antigen challenge” model in which the reporter gene was introduced for the first time at the booster stage. In the settings of “antigen challenge”, we observed a dramatic loss of reporter expression 9 days after the boost, i.e. a week earlier than after a single gene administration. This indicated the pre-existance and boosting of a potent RT-specific immune response that cleared Luc/RT co-expressing cells, which was another agrument advocating for the use of the prime-boost regimen.

Our “antigen challenge” results demonstrated that the prime-boost regimen induced effector/lytic responses against RT/Luc-expressing cells. Such clearance on the background a weak cellular response and strong antibody response against RT, indicates that antibodies play a key role in the clearance process. Indeed, correlation analysis of the loss of bioluminescence with cellular and antibody responses demonstrated a strong direct correlation with anti-RT antibody response (Petkov S. *et al*., PLoS One manuscript [PONE-D-17-40839] - [EMID: 2890cecde7221c73], accepted). One of the plausible mechanisms of the antibody-dependent clearance is the antibody dependent cellular cytotoxicity (ADCC). Interestingly, Pol peptides are common targets of ADCC responses in chronically HIV-1–infected subjects (although these responses decline over time and become ineffective in delaying HIV progression)^112^. Protection against infection with SIV correlates with the induction of ADCC-promoting antibodies specific to the CD4-T-cell epitopes and occurs on the background of a balanced CD4+ T-cell response^37,113^. ADCC contributed to the 31% reduced risk of HIV infection in the RV144 trial^114^, to viral inhibition in the elite controllers^115^, and to protection against infection with SIV and SHIV in rhesus macaques^113,116^. These findings stress the importance of the induction of ADCC for the development of the effective prophylactic as well as therapeutic HIV-1 vaccines. The exact mechanism of immune clearance of RT-expressing cells in RT-DNA immunized mice is currently under study.

The fact that we were able to induce a strong immune response to RT with the effector potential but at the same time were unable to significantly shift the anti-RT immune response from the Th2 to the Th1 type deserves special attention. This reveals the effect of optimizations, of DNA immunogen and of the delivery/immunization regimens, on the terminal outcome of Th polarization. The latter was not pre-defined by the use of DNA immunization modality (predisposing for Th1), nor by the immunogen dose. RT induced a Th2 response after immunization with viral genes that directed low levels of RT expression, while immunization with an overexpressing HIV protease gene induced a strong cellular response but no anti-protease antibodies^30^. Finally, in a parallel study, we have shown that RT induces Th2 responses independently of the immunization route (after both intradermal and intramuscular gene deliveries; Petkov S. *et al*., PLoS One manuscript [PONE-D-17-40839] - [EMID: 2890cecde7221c73], accepted). Thus, Th polarization appears to be defined not by the vaccine vehicle (DNA), immunogen dose, or immunization route, but rather by the intrinsic properties of the immunogen. We demonstrated (here and also in^43^) the secretion of diverse HIV-1 RT variants, with the amounts of extracellular RT equal to that contained within the expressing cells, which turns RT into an exogenous immunogen, available for lysosomal processing and presentation by MHC class II molecules. Further, we have shown that RT induces oxidative stress and oxidative stress responses^42^, both of which promote Th2 polarization^117–120^. Interestingly, two other secretable HIV proteins, Tat and Vpr, also induce oxidative stress^53,121^. The capacity to enter the intercellular space may turn Tat, Vpr, and RT into signaling molecules and contribute to their ability to induce oxidative stress. These RT features are largely insensitive to manipulations with immunization protocols, thus explaining why the Th2 response overrode the Th1 response despite all optimizations.

Importantly, DR mutations had no effect on the overall strength of the cellular immune response, but strongly influenced its nature and specificity. Namely, DR mutations at RT aa 202, 207, 214, and 215 in the context of the virus-derived antigen interfered with the immune recognition of a promiscuous CD4+ T-cell epitopic cluster localized at aa 202–220 that is recognized by the immune systems of mice and humans^80^, as well as recognition of a CTL epitope localized at aa 205–220. Interestingly, only the splenocytes of RTwt (but not of the DR RT) immunized mice recognized both wild-type and drug-resistant variants of the peptide. In other words, in mice, the introduction of DR mutations mimicked the “immune escape”. Indeed, amino acid position 205 of HIV-1 RT lies within an HLA-A*02 cytotoxic T-lymphocyte epitope^122^ and is subjected to the immune escape mutations in subjects with a loss of control over viral replication^100^. The loss of immunogenicity of RT with mutations at aa 202/207/214/215 in HIV-1 patients would then promote the spread of the respective HIV variants that would be dually resistant to the antiretroviral drugs and to the host immune response. This phenomenon motivates an extensive bioinformatics analysis of the frequency of occurrence of the T-cell epitopes in the wild-type versus circulating multidrug-resistant HIV variants. These findings also indicate the necessity to replace the microbial antigens adapted to evade, not induce, anti-viral immunity with the optimally engineered antigens of the desired immune specificity^123^. For vaccines against drug-resistant HIV, one should choose the consensus amino acid sequences with inserted single/multiple primary DR mutations that exclude the imprints of viral adaptation to the host immune response. If the above concept holds true, such genes would be more immunogenic than the synthetic codon-optimized genes encoding the parental viral proteins.

In conclusion, we have systematically optimized the process of DNA immunization to be used with HIV enzyme genes involved in drug resistance, namely HIV-1 RT. For HIV-1 RT, a combination of codon optimization with optimal gene delivery into the skin augmented by electroporation promoted a strong Th2 type immune response manifested by a high level of anti-RT antibodies and a moderate cellular response with effector potential that ensured efficient elimination of expressing cells from the sites of gene administration. Such response may have a direct relation to the success of HIV vaccination. In support of this, we have unpublished data on the capacity of the optimized RT gene immunization to protect mice against the challenge with syngenic adenocarcinoma cells expressing RT protein (Isaguliants M. *et al*., unpublished, Annual Congress of the International Society of Vaccines, Paris, October 5–7, 2017; O7.7 https://isvcongress.org/images/downloads/2017_isv_program.pdf). Both the optimized immunogens/immunization technique and the surrogate pathogen challenge model will be useful for non-human primate trials of the candidate vaccines against drug-resistant HIV.

## Materials and Methods

### Plasmids

This study employed two variants of RT, including RT of HIV-1 HXB2 (RTwt; Gen Bank accession number AAB50259) and RT of HIV-1 HXB2 with multiple drug resistance conferring mutations (RT1.14)^42^. RTwt and RT1.14 genes were subjected to codon optimization (Evrogen, Moscow, Russia) yielding the RTwt-opt and RT1.14-opt gene variants. To increase protein expression, the genes were provided with an AAT-ATG-GGA sequence at their 5′ end resulting in Met-Gly at the protein N-terminus. The enzymatic activities of RTwt-opt and RT1.14-opt were abrogated by site-directed mutagenesis (Evrogen) that introduced mutations D187N and D188N (polymerase activity) and E480Q (RNase H activity), resulting in the RTwt-opt-in and RT1.14-opt-in gene variants. All RT coding sequences were cloned into the eukaryotic expression vector pVax1 (Invitrogen, USA) generating six plasmids – pVaxRTwt, pVaxRTwt-opt, pVaxRTwt-opt-in, pVaxRT1.14, pVaxRT1.14-opt, and pVaxRT1.14-opt-in. Plasmids were purified using Plasmid Endofree kits (Qiagen, Germany) as described by the manufacturer.

### Proteins and peptides

RT proteins used for *in vitro* T-cell tests and ELISAs were purified from *E. coli* cells. The genes of active RT of HIV-1 HXB2 and RT1.14 were cloned into the two-cistron vector pET-2c. Proteins were expressed in *E. coli* and purified by ion exchange chromatography as homodimers using a protocol described earlier^124^. Wild-type p66/p51 heterodimeric HIV-1 RT was expressed in the M-15 [pREP4] *E. coli* strain transformed with the plasmid p6HRT^125^ and purified as previously described^126^. Peptides used in *in vitro* T-cell tests (Table 2) were from GL Biochem Ltd (China).

### RT expression and polymerase activity in eukaryotic cells

All experiments were performed in HeLa cells. Cell cultivation, transfection, and harvesting; the concentration of cell culture fluids; and Western blotting of cell lysates and of the concentrated cell culture fluids were performed as described previously^43^. The Western blotting data were processed in ImageJ software (http://rsb.info.nih.gov/ij). The percent of transfection was estimated in a control co-transfection with GFP plasmid (peGFP-N1, Novagen, Germany). The proportion of RT p66 and p51 subunits in the cell lysates and cell culture fluids was quantified by Western blotting with subsequent ImageJ signal quantification. RT p66 and p51 subunits were quantified separately using calibration curves calculated from the respective RTs loaded on the same gel as the recombinant proteins (with the assumption that anti-RT rabbit antibodies recognize both subunits with similar affinity)^59^. The total amount of expressed RT variant was calculated as the sum of the signals generated by both p66 and p51 subunits in the cell lysates and in cell culture fluids. The relative amount of p51 expressed by the cells was expressed as the p51/p66 ratio. The amount of active RT in the cell lysates and cell culture fluids (irrespective of the subunit structure) was quantified by an RT activity test (Cavidi, Lenti RT Activity Kit, Sweden) and was normalized to the total amount of RT protein as assessed by Western blotting. Data obtained for the lysates or cell culture fluids of a known number of transfected/expressing cells were recalculated per one expressing cell.

### DNA immunization of mice

Eight-week-old BALB/c mice from Charles River Laboratories (Sandhofer, Germany) or Nursary “Pushchino” (Institute of Bioorganic Chemistry RAS, Pushchino, Russia) were housed under a 12 h/12 h light-dark cycle with ad libitum access to water and food. Animals were anesthetized by a mixture of 4% isofluorane with oxygen and maintained with a 2.3% isofluorane flow administered through a facial mask during all intradermal injections and electroporations. Experiments were carried in compliance with the bioethical principles adopted by the European Convention for the Protection of Vertebrate Animals Used for Experimental and Other Scientific Purposes (Strasbourg, 1986) and the Order of the Ministry of Health of the Russian Federation of August 23, 2010, “Establishment of the Rules of Laboratory Practice” No. 708n. Experimental procedures were approved by the local ethics committee of the Federal Research and Clinical Center of Specialized Medical Care and Medical Technologies FMBA of Russia (No. 10a from 09/12/2015) and the Northern Stockholm Ethical Committee for Animal Experiments (permit N66/13).

Groups consisted of 4–8 mice. Each mouse received two intradermal DNA injections delivered to the left and to the right of the base of the tail. In single injections, mice received 10 µg of pVax-based RT-encoding plasmids or empty pVax1 vector mixed 1:1 (w/w) with a pVax-based plasmid encoding firefly luciferase (pVaxLuc) diluted in 20 µl (needle delivery) or 100 µl (Biojector delivery) of PBS per site (standard immunization protocol). In double prime immunizations, the first injection was performed following the standard immunization protocol, and the procedure was repeated on day 5. In prime-boost experiments, mice were primed with 20 µg of RT-encoding plasmids in 20 µl of PBS per site; after 28 days they were boosted according to the standard protocol with 10 µg of pVax-based RT-encoding plasmids mixed 1:1 (w/w) with Luc-encoding plasmid per site. In the set of experiments optimizing the delivery regimen, mice received 20 µg of pVaxLuc in 20 µl of PBS per injection site. Plasmids were administered with 29G-needle insulin syringes, microneedles (Micronjet 600, Nanopass Technologies, Israel), or a Biojector (Biojector©2000, iHealthNet LLC., USA). Injections were followed by electroporation using either a Dermavax DNA vaccine delivery system (Cellectis, Glen Burnie, France) with multi-needle electrodes as described earlier^70^ or a CUY21EditII *in vivo* electroporator (BEX Ltd, Japan) with flat (plate-plate; CUY650-10, P10), fork-plate (CUY663-5 × 10), two-needle (CUY560-5), and multi-needle (CUY691-5) electrodes. The electrodes were applied with the following combinations of pulse parameters: a poration pulse of 400 V (lasting for 0.05 ms with a 50 ms gap) followed by 8 driving pulses of 10 ms or 50 ms performed at 50 V or 100 V. Pulses were delivered with 20 ms (in case of 10 ms pulse) or 950 ms (in case of 50 ms pulse) gaps with eitherconstant (+/+) or opposing (+/−) polarity. Immunization with the c-di-GMP adjuvant was performed in the frame of the double prime regimen. C-di-GMP (1 nmol; Sigma, USA) diluted in 100 µl of PBS was administered intraperitoneally at the time of the RT-gene immunizations. On day 15 after the first injections, mice were bled to assess the immune response of PBMCs. On day 21 after the single injection or on day 21 after the second injection (in the double prime and prime-boost regimens), mice were bled, sacrificed, and their spleens were collected for further immune assays. Details of all immunization regimens are provided in the legends of the figures illustrating the results.

### Analysis of cellular response to RT and luciferase in PBMCs and splenocytes by IFN-γ ELISpot and IFN-γ/IL-2 Fluorospot

The PBMCs and splenocytes of immunized mice were isolated as described in^31^. PBMCs were pooled group-wise. The cells were incubated in RPMI medium supplemented with 2 mM L-glutamine, 2 mM penicillin-streptomycin, and 10% FBS (Gibco, Invitrogen) (complete media). The antigens described in the section “Proteins and peptides” were used at concentrations of 10 µg/ml; concanavalin A was used at a concentration of 5 µg/ml as a positive control and cell culture medium served as a negative control. After 20 hours of incubation, IFN-γ secretion by PBMCs or IFN-γ and IL-2 secretion by splenocytes was assessed in IFN-γ ELISpot or dual IFN-γ/IL-2 Fluorospot tests (Mabtech, Sweden) in accordance with the protocols provided by the manufacturer. The number of cells secreting cytokines was counted on an AID ELISpot fluorimeter (Autoimmun Diagnostika GmbH, Germany).

### Analysis of the cellular response to RT in splenocytes by flow cytometry with intracellular cytokine staining

All of the procedures were performed as previously described in^109^. Briefly, splenocytes of RT-immunized or vector-immunized mice were stimulated with RT-derived peptides RT202–210 wt, RT202–210 dr, RT207–215 wt, RT207–215 dr, RT205–220 wt, and RT205–220 dr (Table 2) and stained for viability with the Fixable Viability Stain 660 (FSV660; BD Horizon #564405). Thereafter, cell surface staining was performed with a mixture of antibodies including FITC-conjugated anti-mouse CD8a (BD Pharmingen #553031), APC-H7-conjugated anti-mouse CD4 (BD Pharmingen #560181), and PerCP-conjugated anti-mouse CD3 (BD Pharmingen #553067). The cells were then fixed, permeabilized, washed, and stained with PE-Cy7-conjugated anti-mouse IFN-γ antibodies (BD Pharmingen #557649). Stained samples were analysed on a FACSVerse cytometer (BD Biosciences, USA). Data were exported as FCS3.0 files with the use of FACSuite software and read using BioConductor’s^127^ package flowCore^128^ in the R software package. Final normalization of the data was performed with the flowStats package^129^ and the data were gated. First, a general lymphocyte area was defined and viable cells were identified by the lack of FSV660 staining. From the viable population, single cells were defined by the expression of surface markers and IFN-γ production.

### Analysis of anti-RT-specific humoral response by indirect ELISA

ELISA was carried on MaxiSorb 96-well plates (Nunc, USA) coated overnight at 6 °C with the recombinant RTwt-opt or RT1.14-opt proteins at 0.3 mg/ml in PBS. All ELISA experiments were performed as described previously^43^. The cut-off for specific RT antibody response was set as the mean optical density (OD) values shown by the sera of vector-immunized mice at this time point +3 SD. For positive sera showing OD values exceeding the cut-off, endpoint dilution titers were established from the titration curves.

### *In vivo* imaging of luciferase expression

Bioluminescence emission from the area of DNA-immunogen/Luc gene injection was analysed on days 1, 3, 9, 15, and 21 after the prime or boost immunizations. The detailed protocol was described in^43^. In brief, the *in vivo* imaging of bioluminescence was performed with a highly sensitive CCD camera mounted in a light-tight chamber (Lumina, or IVIS Spectrum CT, Perkin Elmer, USA). Anesthesia was induced by 4% isofluorane and maintained by 2.3% isofluorane throughout the imaging procedure. Ten minutes prior to capturing of the luminescent signal, mice were injected intraperitoneally with a solution of D-luciferin (Perkin Elmer #122796, USA) in PBS at a dose of 150 mg/kg body weight. The animals were monitored on days 1, 3, 9, 15, and 21 after the reporter gene injections. Regions of interest were localized around the injection sites and were quantified as the total photon flux (photons/s) and the average photon flux per square centimeter (photons/s/cm^2^/sr) demonstrating the number of photons per second leaving a square centimeter of tissue and radiating into a solid angle of one steradian (sr). Bioluminescence imaging data was processed using the Living Image® software version 4.2 (Perkin Elmer, USA).

### Statistics

Statistical analyses were performed using STATISTICA AXA 10.0 (StatSoft Inc., OK, USA). Non-parametric statistics were chosen as appropriate for sample sizes with fewer than 100 entries. Continuous but not normally distributed variables, such as the average radiance in photons/s/cm^2^/sr, antibody titers, or the number of cytokine-producing spot forming cells, were compared in groups by the nonparametric Kruskal-Wallis and pair-wise by the Mann-Whitney U-test. P-values < 0.05 were considered significant.

### Data availability

All data generated or analysed during this study are included in this published article and its Supplementary Information files.

## Electronic supplementary material

Supplementary material
