# Supplementary material for: Codon optimization and improved delivery/immunization regimen enhance the immune response against wild-type and drug-resistant HIV-1 reverse transcriptase, preserving its Th2-polarity
Source: Sci Rep. 2018 May 24;8:8078. doi: 10.1038/s41598-018-26281-z (PMC5967322; doi:10.1038/s41598-018-26281-z)
Supplement: Supplementary file 1 — Supplementary material [file 41598_2018_26281_MOESM1_ESM.docx]

**Codon optimization and improved delivery/immunization regimen enhance the immune response against wild-type and drug-resistant HIV-1 reverse transcriptase preserving its Th2-polarity**

Latanova A.A. 1,2,3^#*^, Petkov S. 2^#^, Kilpelainen A. 2, Jansons J. 5, Latyshev O.E. 3,4, Kuzmenko Y.V.1, Hinkula J.2,6, Abakumov M.A^7,8^, Valuev-Elliston V.T. 1, Gomelsky M. 9, Karpov V.L. 1, Chiodi F.2, Wahren B.2, Logunov D.Y. 3,4, Starodubova E.S.1,4^##^, Isaguliants M.G. 3,4,5^##*^

1. Engelhardt Institute of Molecular Biology, Russian Academy of Sciences, Moscow, Russia;
2. Department of Microbiology, Tumor and Cell Biology, Karolinska Institute, Stockholm, Sweden;
3. Gamaleja Research Center of Epidemiology and Microbiology, Moscow, Russia;
4. Chumakov Federal Scientific Center for Research and Development of Immune-and- Biological Products of the Russian Academy of Sciences, Moscow, Russia;
5. Riga Stradins University, Riga, Latvia
6. Linköping University, Linköping, Sweden
7. Research and Education Center for Medical Nanobiotechnology, Pirogov Russian National Research Medical University, Ministry of Health of the Russian Federation, Moscow, Russia
8. National University of Science and Technology (MISIS), Moscow, Russia
9. Department of Molecular Biology, University of Wyoming, Laramie, WY, 82071, USA

#- shared first authorship

## - shared last authorship

* - corresponding authors, Anastasia Latanova, [aalatanova@gmail.com](mailto:aalatanova@gmail.com); Maria Isaguliants, [maria.issagouliantis@rsu.lv](mailto:maria.issagouliantis@rsu.lv)


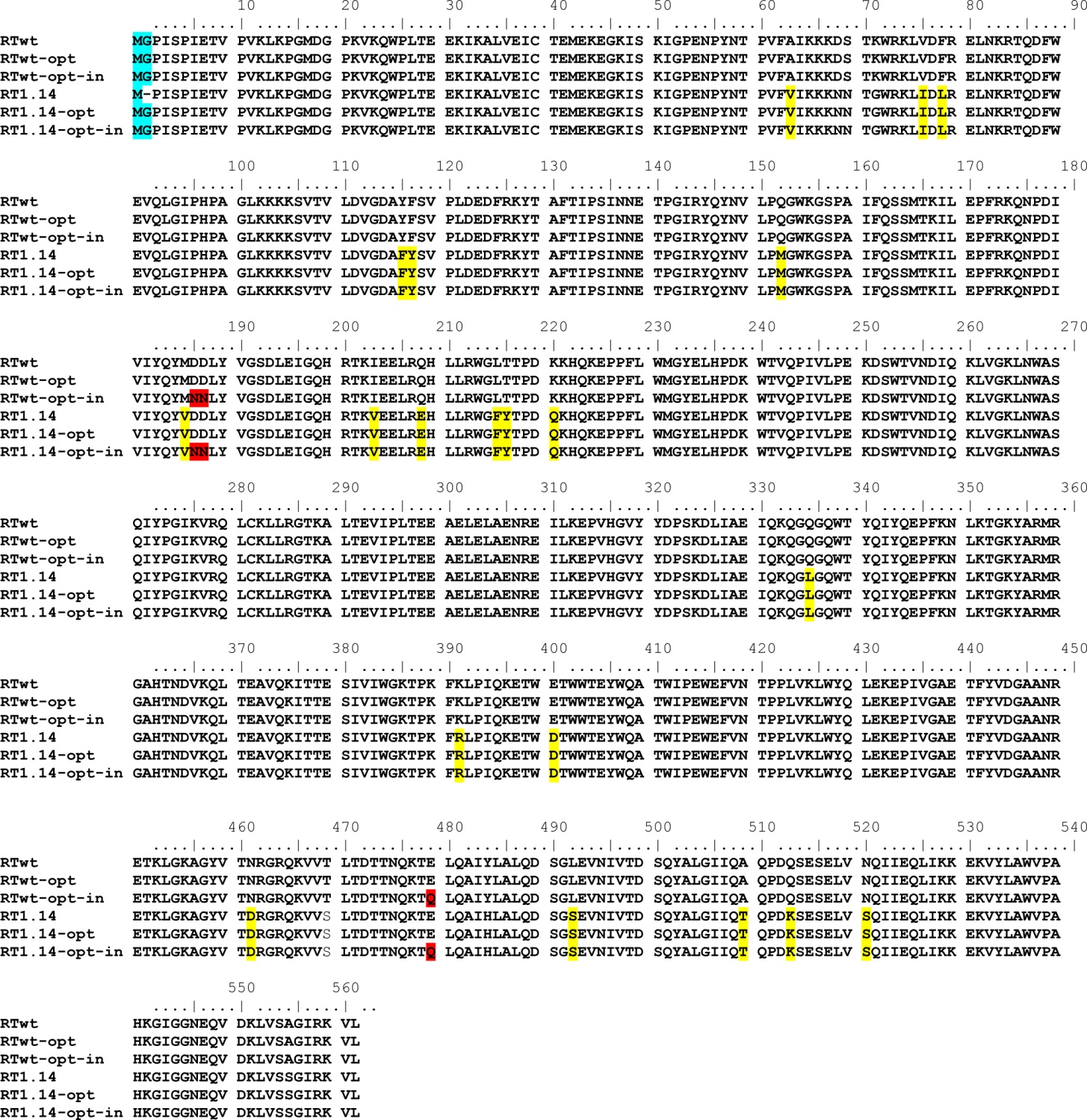


*Supplementary Figure S1.* *Alignment of amino acid sequences of RT HIV-1 strains HXB2 (RTwt) and MN with multiple drug resistance conferring mutations (RT1.14) encoded by viral and expression-optimized synthetic genes of active (RTwt-opt; RT1.14-opt) and inactivated enzymes (RTwt-opt-in; RT1.14-opt-in). Mutations A62V/D67N/S68N/K70G/V75I/F77L/I115F/F116Y/Q151M/M184V/T215Y/K219Q occurring in RT1.14 confer multiple resistance to NRTI* [^1^](#_ENREF_1). *The N-terminal Met-Gly translated from the Kozak sequence are designated in blue; drug resistance conferring mutations in yellow; mutations abrogating polymerase and RNase H activities, in red.*





*Supplementary Figure S2. Expression of RT variants in eukaryotic cells.* *Western blotting of the lysates of HeLa cells transfected with vector pVax1 (lane 1), and pVax-based plasmids expressing RTwt (lane 2 a, c, e), RTwt-opt (lane 3 a, c, e), RTwt-opt-in (lane 4 a, c, e), RT1.14 (lane 3 b, d, f), RT1.14-opt (lane 4 b, d, f), and RT1.14-opt-in (lane 5 b, d, f), or with pKCMVRT1.14 (lane 2 b, d, f) used in previous experiments*[*^2^*](#_ENREF_2) *(a, b, c, d, e, f). Blots of RTwt and RT1.14 variants were processed in parallel. Blots were stained with rabbit polyclonal anti-RT antibodies*[*^3^*](#_ENREF_3) *(a-d) and then stripped and re-stained with monoclonal anti-actin antibodies (e, f). Positions of the relevant molecular mass markers (Page Ruler Prestained Protein Ladder, Thermo Scientific) are given to the right in kDa. Arrows point at the p66 and p51 RT subunits. Panels (a, b) represent results of a 30 s exposure of X-ray film with a blot; panels (c, d) represent results of a 10 min exposure.*







(e)

*Supplementary Figure S3. Accumulation and polymerase activity of multidrug resistant RT1.14 variants in transfected cells and culture fluids. Western blotting of the lysates (lanes 1, 2) and cell culture fluids (lanes 3, 4) of HeLa cells transfected with pVax1 vector (lanes 1, 4) and pVaxRT1.14opt-in (lanes 2, 3) (a, b). The cell lysates and cell culture fluids were processed on one blot (full-length blots are presented on (c, d)). Rabbit polyclonal antibodies were used to stain for RT*[*^3^*](#_ENREF_3) *(a). The blots were then stripped and re-stained with monoclonal anti-actin antibodies (b). Positions of the relevant molecular mass markers (Page Ruler Prestained Protein Ladder, Thermo Scientific) are given to the right in kDa. The full-length Western blotting (presented in its cropped version on (a, b)) of the lysates (lane 1-5) and cell culture fluids (lane 7-9) of HeLa cells transfected with pVax1 vector (lane 1, 9), pVaxRT1.14opt-in (lane 2, 7), and pVaxRT1.14oil (lane 3-5, 8, described in*[*^4^*](#_ENREF_4)*) (c, d); anti-RT staining (c), anti-actin staining (d). Residual enzymatic activity of RT1.14 in transfected HeLa cells and cell culture fluids (e). The specific RT activity was measured as a ratio of protein content in one expressing cell evaluated by Cavidi test (in fg multiplied by 10^3^) to RT content determined by Western blotting (in pg).**p<0.01 when compared to RT1.14 encoded by the viral gene. Statistical comparisons were performed using Mann-Whitney tests.*

(a) (b)

(c) (d)

**

*Supplementary Figure S4. In vivo monitoring of bioluminescence in mice co-injected with RT gene variants and luciferase (Luc) gene.* *BALB/c mice (n=6) were immunized with two intradermal injections (29G needle) containing 20 µg of RTwt, RTwt-opt, RTwt-opt-in, RT1.14, RT1.14-opt, and RT1.14-opt-in encoding plasmids per mouse mixed 1:1 (w/w) with a Luc-encoding plasmid and subsequent electroporation by Dermavax (standard protocol) in two independent immunization runs. Control mice received a Luc gene mixed with pVax1. Monitoring of bioluminescence was performed on days 1, 3, 9, 15, 21 after the immunization. Each curve (a, b) or bar (c, d) represents an average photon flux (photons/s/cm^2^/sr) from the injection area observed for the group of six mice (12 immunization sites), and the error bars represent the SD. Dynamics of bioluminescence at the sites of co-injection of a Luc gene and RT variants based on RTwt genes (RTwt, RTwt-opt, and RTwt-opt-in) (a,c), and RT.14 genes (RT1.14, RT1.14-opt, and RT1.14-opt-in) (b,d).The difference in photon flux between RT- and vector immunized mice (a, b) and between groups of mice receiving different RT gene variants (c, d) are indicated by asterisk; ∗, p<0.05; ** p<0.01. Statistical comparisons were performed using Mann-Whitney tests.*

(a)

(b) (c) (d) (e)

*Supplementary Figure S5. Optimization of gene delivery by application of 29G needles, microneedles and needle-free devices with electroporation using penetrating and non-penetrating electrodes, defined by in vivo monitoring of Luc expression.* *BALB/c mice (n=6–8) were immunized with two intradermal injections of 20 µg of RT1.14-opt-in encoding plasmid per mouse mixed 1:1 (w/w) with a plasmid encoding Luc using an insulin syringe with a 29G needle (29G), microneedles Micronjet 600 (Microneedle), or a Biojector 2000 (Biojector) and electroporated using Dermavax with multi-needle electrodes (Dermavax-MN) or injected with a mixture of Luc/RTwt-opt-in encoding plasmids using an insulin syringe with a 29G needle and electroporated with the BEX machine and flat electrodes (29G-BEX-FL).The level of Luc expression assessed on days 1, 3, 9, 15, 21 and presented as the ratio between an average bioluminescence and the highest achievable level of bioluminescence from the injection areas in each group, and the error bars represent the SD (a); average bioluminescence from all the injection areas in mice co-immunized with Luc/RT1.14opt-in (b, c), or Luc/empty vector (d, e) on days 1 (b, d) and 3 (c, e) after the immunization. *p<0.05, **p<0.01. In (a) in case of Biojector-Dermavax-MN and 29G-BEX-FL regimens the asterisks designate their difference from both 29G-Dermavax-MN and Microneedle-Dermavax-MN regimens. Statistical comparisons were performed using Kruskal-Wallis and Mann-Whitney tests.*


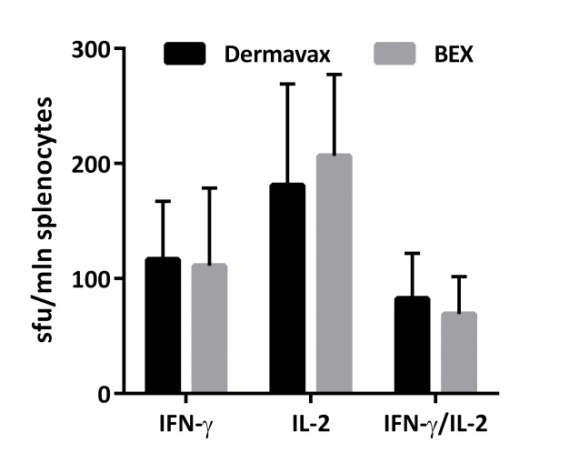


*Supplementary Figure S6. Comparison of specific cellular immune response against the RT gene delivered by intradermal injections with electroporation by BEX or Dermavax devices. BALB/c mice (n=6) were immunized with two intradermal injections with 20 µg of RTwt-opt-in encoding plasmid per mouse and electroporated using Dermavax or BEX with multi-needle electrodes. At 21 days post immunization, mice were sacrificed and splenocytes were isolated and subjected to in vitro stimulation with peptide representing aa 528-543 of HIV-1 RT, and cytokine secretion was assessed by dual IFN-*γ*/IL-2 Fluorospot. The bars represent the average number of cells, registered as signal-forming units (sfu) per million splenocytes secreting IFN-*γ*, IL-2, and IFN-*γ*/IL-2, and the error bars represent the SD. All assays were performed in duplicate. No difference (p>0.1) was observed between the immune responses after electroporation with Dermavax and BEX. Statistical comparisons were performed using Mann-Whitney tests.*

***
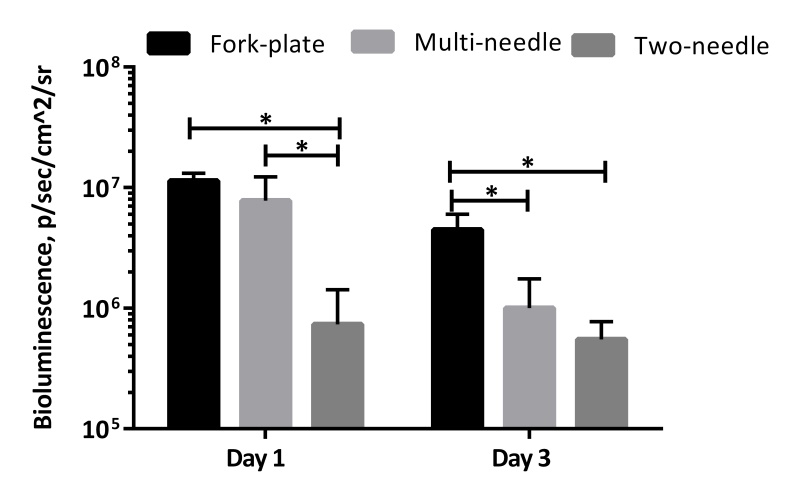
***

*Supplementary Figure S7. Comparison of luciferase expression after electroporation using fork-plate, multi-needle, and two-needle electrodes. BALB/c mice (n=4) were intradermally injected at two sites with 20 µg Luc per mouse, and immediately electroporated with the BEX machine equipped with the fork-plate, multi-needle and two-needle electrodes. Bioluminescence was monitored by in vivo imaging on day 1 and 3 after the injections. The data represent the average photon flux from all injections sites in the group (photons/s/cm^2^/sr), and the error bars represent the SD. *p<0.05. Statistical comparisons were performed using Mann-Whitney tests.*

**REFERENCES**

1 Jonckheere, H., Anne, J. & De Clercq, E. The HIV-1 reverse transcription (RT) process as target for RT inhibitors. *Medicinal research reviews* 20, 129-154 (2000).

2 Isaguliants, M. G. *et al.* Mutations conferring drug resistance affect eukaryotic expression of HIV type 1 reverse transcriptase. *AIDS research and human retroviruses* 20, 191-201, doi:10.1089/088922204773004914 (2004).

3 Isaguliants, M. G. *et al.* Immunogenic properties of reverse transcriptase of HIV type 1 assessed by DNA and protein immunization of rabbits. *AIDS research and human retroviruses* 16, 1269-1280, doi:10.1089/08892220050117032 (2000).

4 Latanova, A. *et al.* Fusion to Flaviviral Leader Peptide Targets HIV-1 Reverse Transcriptase for Secretion and Reduces Its Enzymatic Activity and Ability to Induce Oxidative Stress but Has No Major Effects on Its Immunogenic Performance in DNA-Immunized Mice. *Journal of immunology research* 2017, 7407136, doi:10.1155/2017/7407136 (2017).
